# Structural stability-guided scaffold hopping and computational modeling of tankyrase inhibitors targeting colorectal cancer

**DOI:** 10.1371/journal.pone.0332798

**Published:** 2025-09-19

**Authors:** Mohammed Merae Alshahrani

**Affiliations:** Department of Clinical Laboratory Sciences, Faculty of Applied Medical Sciences, Najran University, Najran, Saudi Arabia; University of Mashreq, IRAQ

## Abstract

Colorectal cancer is one of the leading causes of cancer-related deaths worldwide, mainly due to aberrant Wnt/β-catenin signaling resulting from APC mutations. Tankyrase is a key regulator of this pathway and plays a crucial role in stabilizing AXIN, a negative regulator of β-catenin, and hence an attractive therapeutic target. The present study describes a comprehensive computational approach to discovering novel tankyrase inhibitors for CRC therapy. The reference (RK-582) for ligand-based screening and comparative analysis was taken from the crystal structure of tankyrase. A similarity search in the PubChem, applying an 80% cutoff, yielded 533 structurally similar compounds. These compounds were subjected to virtual screening using a drug-likeness filter. The top-ranking binding poses of three selected compounds (PubChem CIDs: 138594346, 138594730, and 138594428) were selected for DFT calculation and re-docking. DFT calculations revealed that compound 138594428 had the largest HOMO-LUMO gap (4.979 eV), indicating high electronic stability, while 138594346 exhibited a strong balance of stability and reactivity (4.473 eV). The MD simulations were conducted on all ns protein-ligand complexes for 500 ns, exploring their stability. MD simulations confirmed the conformational stability of these compounds, with 138594346 showing the lowest RMSD and RMSF fluctuations. Additionally, a machine learning model trained on 236 known Tankyrase inhibitors accurately predicted pIC₅₀ values, with compound 138594346 (pIC₅₀ = 7.70) closely matching the reference inhibitor (pIC₅₀ = 7.71), and 138594428 also exhibiting strong predicted activity (pIC₅₀ = 7.41). Collectively, these results highlight 138594346 and 138594428 as promising candidates for further experimental validation in the development of targeted CRC therapeutics.

## Introduction

Colorectal cancer (CRC) is one of the most important causes of morbidity and mortality due to cancer worldwide. It is the third most common cancer and the second most lethal among all cancer cases [1,2]. GLOBOCAN 2022 reported more than 1.9 million new cases of CRC and about 904,000 deaths around the world [3,4]. While CRC has traditionally been considered a disease of older individuals, incidence rates have increased substantially over the past two decades for younger populations; early-onset CRC is also commonly diagnosed at more advanced stages, with aggressive histological features, compared to later stages in life [4,5]. Obesity, physical inactivity, and a high intake of processed foods and red meat were suggested as major risk factors for this trend, adding to genetic predispositions like Lynch syndrome and familial adenomatous polyposis [6–8].

CRC develops via a series of genetic and epigenetic changes, generally described by the adenoma-carcinoma sequence [9]. Some key genes driving the progression of CRC include adenomatous polyposis coli, KRAS, and TP53. The mutation of APC is highly critical because this gene product participates in the Wnt/β-catenin signaling pathway, a key modulator of cell proliferation and differentiation. The disruption of this pathway results in nuclear accumulation of β-catenin, driving tumor growth and survival [10–12].

Tankyrase represents a PARP with fundamental roles in modulating the Wnt/β-catenin pathway. It promotes the ubiquitination and subsequent degradation of AXIN, a critical scaffold protein of the β-catenin destruction complex [13]. Treating cells with inhibitors against tankyrases stabilizes AXIN, increases β-catenin degradation, and suppresses Wnt signaling. For this reason, from a therapeutic point of view, tankyrase is one of the most attractive targets, especially in those APC-mutated CRCs harboring hyperactivated Wnt signaling [14,15].

Preclinical studies have validated the efficacy of tankyrase inhibitors through selective action on CRC cells with minimal typical cell effects [16]. However, in clinical use, agents based on such mechanisms have been plagued by some drawbacks in off-target effects and gastrointestinal toxicities. These are all challenges that indicate the need for the development of novel selective inhibitors with an improved pharmacological profile [17].

These new developments in the computational techniques of drug discovery enable a new therapeutic agent to be identified much faster [18]. The whole process helps prioritize the compounds for experimental evaluation so that one can focus on the complexes that present favorable interactions. Molecular dynamics simulations can further provide details regarding the stability and dynamics of protein-ligand interactions in physiological conditions. Scaffold hopping is considered to be one of the key instruments of lead optimization because it gives a way of finding a new chemical scaffold possessing better properties while conserving critical pharmacophoric features [19–21].

Drug discovery approaches have significantly facilitated the pursuit of novel tankyrase inhibitors. Among them, the PubChem database is an invaluable resource for such studies [22]. Maintained by the National Center for Biotechnology Information, PubChem is one of the largest repositories of chemical information, containing over 100 million unique compounds. It offers extensive chemical structure, properties, and biological activity data and enables advanced searches based on structural similarity, substructure, and molecular properties.

In the present study, I used the PubChem database in the search for CRC-treating tankyrase inhibitors. The compounds were virtually screened and docked into the active site, calculating binding affinities and poses. Quantum chemical calculations were also performed for identified molecules. Then, stability in protein-ligand interactions was analyzed using MD simulations. Finally, a machine learning approach was applied to predict the biological activity of identified compounds, which laid the foundation for further experimental validation in developing effective tankyrase inhibitors.

## Methodology

**Fig 1:**
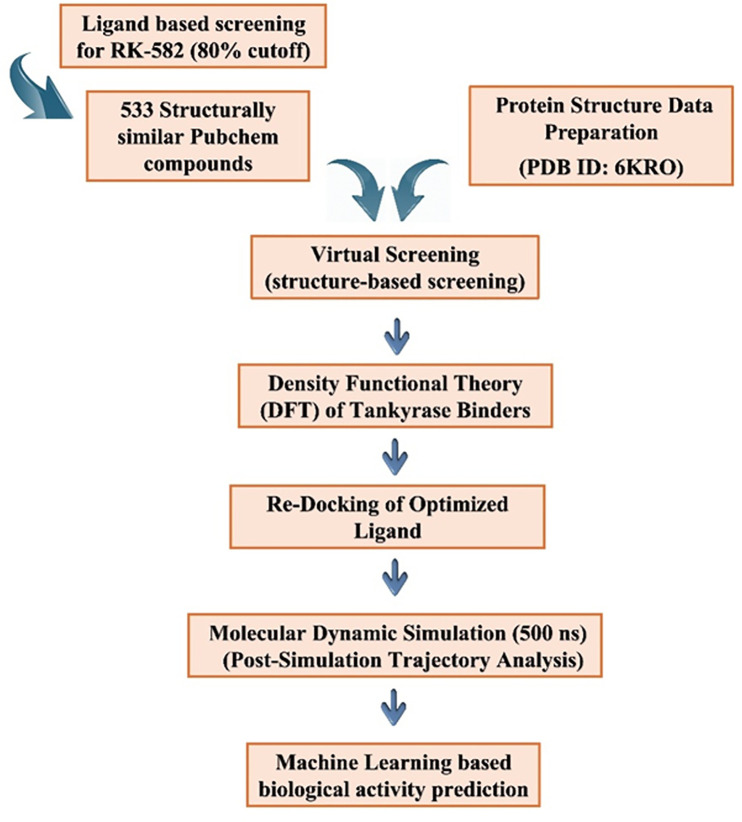
Schematic representation of the integrated computational workflow. The process includes database screening, molecular docking, DFT, MD simulations, and machine learning (ML)-based activity prediction from a systematic pipeline for identifying and prioritizing novel tankyrase inhibitors.

Systematic identification of effective tankyrase inhibitors was carried out through a multi-step computational methodology. The workflow involved ligand-based and structure-based virtual screening, docking of molecules, quantum calculation based on DFT, dynamic stability through MD simulations, as well as biological activity prediction through machine learning. Fig 1 gives a visual overview of the pipeline used.

### Protein structure data preparation

The target protein with a high-resolution 3-D structure freely available in a public depository called the Protein Data Bank (PDB) was selected based on high resolution (1.90 Å) [23]. Therefore, the identified PDB format (PDB ID: 6KRO) file download and its preprocessing were performed using multi-functional software called UCSF Chimera [24,25]. Crystallographic water molecules not participating in the binding of the ligand and existing were removed; the protein was protonated at physiological pH 7.4. Optimization of hydrogen-bond networks and assignment of charges were included to ensure stability and accuracy in subsequent docking and simulation studies. This preprocessing step rendered the protein structure suitable for virtual screening, docking, and molecular dynamics simulation studies.

### Compound library preparation

A reference compound (RK-582) bound to the tankyrase was identified in the retrieved PDB structure and used as a template for similarity screening. Structural similarity searching using the PubChem database, with the similarity cutoff at 80%, was carried out after that to probe chemical space [22]. Consequently, the related structures were retrieved and downloaded as an SDF file. Therefore, energy minimization was performed, and the docking preparations were completed. This puts the compounds in an optimal geometrical form suitable for subsequent virtual screening and computational analysis.

### Density functional theory analysis of tankyrase binders

Density Functional Theory (DFT) calculations were performed using the PySCF quantum chemistry library to investigate the frontier molecular orbitals of selected Tankyrase inhibitor candidates [26,27]. Molecular structures were first extracted from SDF files using RDKit, and hydrogen atoms were retained for accurate geometry representation. Atomic coordinates were parsed and used to define molecular systems for PySCF calculations [28,29]. Each molecule was modeled using the cc-pVDZ basis set and the B3LYP exchange-correlation functional within a restricted Kohn–Sham (RKS) framework. After self-consistent field (SCF) convergence, the molecular orbital coefficients were used to generate three-dimensional cube files of the HOMO and LUMO orbitals. The energies of the HOMO and LUMO orbitals were extracted directly from the eigenvalues of the Kohn–Sham matrix, and the HOMO-LUMO energy gap was computed and reported in electronvolts (eV).

This approach enabled both visual and quantitative comparison of the electronic properties relevant to the compounds’ potential as Tankyrase inhibitors.

### Redocking of optimized ligands

The DFT optimized compounds were further selected for redocking validation. The analysis of the docking simulations was performed with the AutoDock Vina plugin within the Chimera interface [30]. That way, detailed views about the binding orientations and the interaction properties of the selected compounds within the target protein could be obtained. Key stabilizing interactions comprising hydrogen bonds, hydrophobic contacts, and π-π stacking were identified. Redocking was done using the same docking protocol to validate the initial results and ensure consistency. The docking experiments also included a reference molecule of known activity to benchmark the selected compounds’ binding scores and interaction patterns [31].

### ADMET

The ADMET profiles of the selected compounds were predicted using ADMETlab 2.0 (https://admetmesh.scbdd.com), which employs a multi-task graph attention model trained on ~250,000 molecules [32]. Absorption parameters included aqueous solubility, Caco-2 permeability, human intestinal absorption, and oral bioavailability. Distribution properties such as plasma protein binding, volume of distribution, blood–brain barrier permeability, and unbound fraction were assessed. Metabolic and excretion-related features were predicted through cytochrome P450 interactions, total and renal clearance, and biological half-life. Toxicity endpoints included AMES mutagenicity, hERG inhibition, hepatotoxicity, and acute oral toxicity (LD₅₀).

### Molecular dynamics simulations

Tankyrase, a member of the poly(ADP-ribose) polymerase (PARP) family, contains multiple functional domains, including ankyrin repeat clusters, a sterile alpha motif (SAM), and a catalytic PARP domain responsible for ADP-ribosylation. The catalytic domain of human Tankyrase 2, as represented in the crystal structure PDB ID: 6KRO, features a conserved NAD ⁺ -binding pocket that accommodates both substrate and inhibitor molecules. Key active-site residues include Gly1032 and Ser1068, which are commonly involved in hydrogen bonding interactions, along with Phe1035, Tyr1071, and His1048, which contribute hydrophobic and π–π stacking interactions. These residues are known to play essential roles in selective inhibitor recognition and were preserved during simulation setup. Molecular dynamics simulations were subsequently performed to assess the stability and dynamic behavior of the protein–ligand complexes over time using the free academic version of Desmond. These were performed with the protocol in Bowers et al., 2006, and Schrödinger Release, 2024−2, 2024 [33,34].

Desmond-Maestro was used in the preprocessing step using its Protein Preparation Wizard to correct the missing residues, optimize the networks of hydrogen bonds, and assign charges in protein-ligand complexes [33 ]. Afterward, each complex was placed separately in a rectangular box filled with TIP3P water, having 10 Å of buffer on every side to maintain realistic surrounding conditions [35]. Sodium and chloride ions were neutralized at physiological ionic strength, −0.15 M concentration. The OPLS_2005 force field parameterized the complexes [36]. Steric clashes were removed, and system stability was ensured by performing energy minimization for 100 ps. Equilibration was performed in two steps: first, in the NVT ensemble, to stabilize the temperature at 300 K, and second, in the NPT ensemble, equilibrating the system under a constant pressure of 1 atm.

A 500 ns production run was made under the ensemble class of NPT. The Nose-Hoover thermostat was used to sustain the system at a constant temperature, while a Martyna-Tobias-Klein barostat performed the same duties for the pressure [37]. It utilized a RESPA integrator by dividing the operation into multi-timestep sections for bonds and nonbonds to increase computational throughput. Long-range electrostatics were computed at every step using the method of particle mesh Ewald (PME) [38], whose Coulombic and Van der Waals interactions were kept at a 9.0Å cutoff [39].

Trajectory snapshots were saved every 10 ps, which provided 10,000 frames for each complex in case of post-simulation analysis. Post-Simulation Analysis Trajectory analyses included the calculation of RMSD and RMSF as indicators of complex stability and flexibility. The general profile of hydrogen bonding pattern, hydrophobic interaction, and other stabilizing interactions in these complexes was checked through Desmond’s simulation in-built interaction diagram tool.

### Machine learning methodology for pIC₅₀ prediction of tankyrase inhibitors

To develop a predictive model for estimating the pIC₅₀ values of Tankyrase-2 inhibitors, a machine learning-based workflow was implemented using Python and Jupyter Notebooks [27,40]. The approach integrated cheminformatics tools (RDKit) and machine learning libraries (Scikit-learn, XGBoost) to support data preprocessing, feature generation, model training, and evaluation [28,29]. A dataset of 236 Tankyrase inhibitors was retrieved from the ChEMBL database, curated to remove duplicate entries, and standardized using canonical SMILES. Molecular descriptors were generated using both PubChem fingerprints, which capture binary substructure presence, and four key Lipinski descriptors (molecular weight, LogP, hydrogen bond donors, and acceptors) computed via RDKit [29,41].

The dataset underwent Exploratory Data Analysis (EDA) to assess descriptor distributions, remove invariant features, and evaluate correlations. A total of forty regression algorithms were benchmarked, each trained and validated using five-fold cross-validation. Model performance was evaluated using the coefficient of determination (R²), Root Mean Square Error (RMSE), and Mean Absolute Error (MAE). Feature importance scores were computed for tree-based models to identify key predictors. Hyperparameter tuning was performed using grid search to optimize predictive accuracy. The best-performing model was selected based on cross-validation results and retrained on the full dataset to enable robust pIC₅₀ prediction for novel Tankyrase-2 inhibitors.

## Results

### Virtual screening

**Fig 2:**
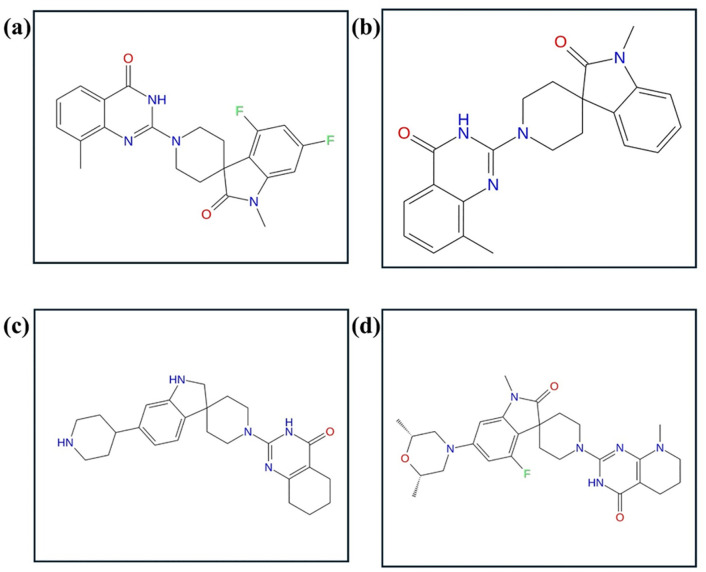
Structure of selected compounds after virtual screening (a) 138594346, (b) 138594730, (c) 138594428, and (d) reference.

A reference compound bound to tankyrase in its crystal structure served as a template in ligand-based structural similarity screening. A similarity cutoff of 80% was used in the PubChem database and resulted in the retrieval of 533 structurally similar compounds. These compounds were subjected to virtual screening against tankyrase. Binding energies for these compounds ranged from −15.0 kcal/mol to −4.0 kcal/mol (S1 Table). Three top-ranked compounds (PubChem CID: 138594346, 138594730, and 138594428) with the best docking scores and one Reference/control compound were selected and further analyzed in detail (Fig 2).

### Quantum chemical evaluation of tankyrase binders

**Fig 3:**
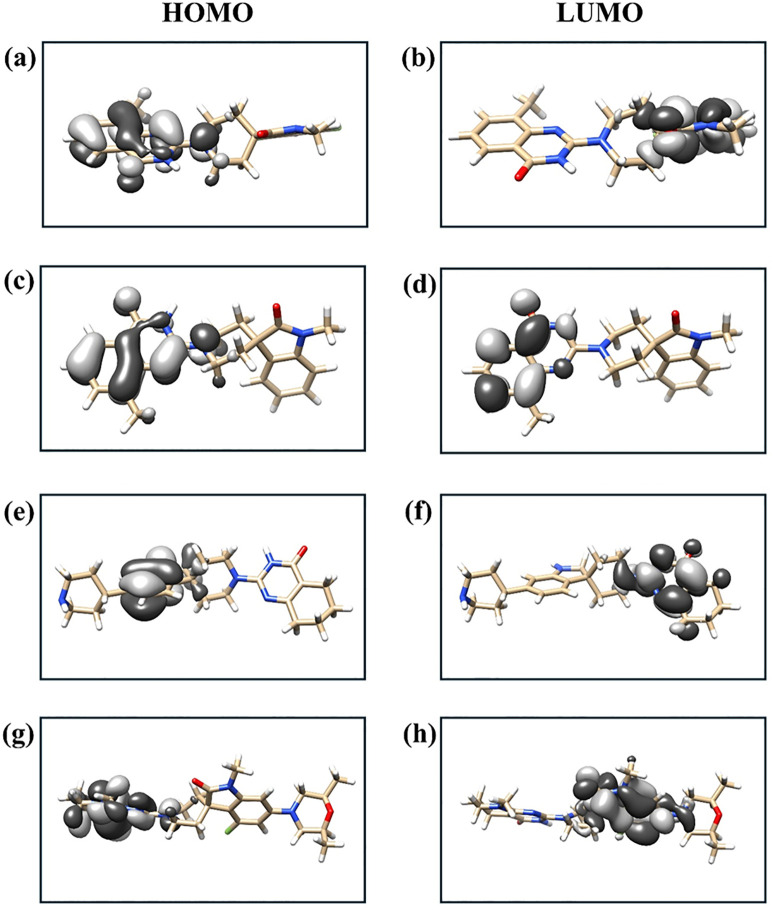
Frontier molecular orbitals (HOMO and LUMO) of top three virtually screened Tankyrase inhibitors and the reference compound. (a-b) 138594346, (c-d) 138594730, (e-f) 138594428, and (g-h) reference.

The Density Functional Theory (DFT) analysis of these four compounds targeting the Tankyrase protein provides valuable insight into their electronic properties, particularly the energy differences between the Highest Occupied Molecular Orbital (HOMO) and the Lowest Unoccupied Molecular Orbital (LUMO). Among the virtually screened candidates, compound 138594346 (Fig 3 (a-b)) exhibits a HOMO energy of −0.202297 Hartree and a LUMO energy of −0.037932 Hartree, resulting in a HOMO-LUMO gap of 4.473 eV. This moderate gap suggests a balanced electronic reactivity, which could contribute to effective interactions with the target protein.

Compound 138594730 (Fig 3 (c-d)) demonstrates a slightly smaller HOMO energy of −0.201801 Hartree and a LUMO energy of −0.029403 Hartree, leading to a HOMO-LUMO gap of 4.691 eV. This indicates a slightly higher electronic excitation potential than 138594346, implying a relatively more stable electronic configuration and potentially better resistance to oxidative degradation.

The third virtually screened compound, 138594428 (Fig 3 (e-f)), shows a HOMO energy of −0.195365 Hartree and a LUMO energy of −0.012392 Hartree. It possesses the most enormous HOMO-LUMO gap of 4.979 eV among the screened compounds, indicating the highest electronic stability and the lowest polarizability. Such characteristics can benefit specificity in molecular interactions, though they might affect binding kinetics.

In comparison, the reference or control compound (Fig 3 (g-h)) has a HOMO energy of −0.164069 Hartree and a LUMO energy of −0.022958 Hartree, giving a HOMO-LUMO gap of 3.840 eV. This is significantly lower than those of the top-screened candidates, suggesting that the reference compound is more electronically reactive and potentially less stable. The higher reactivity might enhance specific interactions and imply reduced specificity or stability.

### Redocking validation

The DFT-optimized ligands were further redocked into the target protein at the same site. The top three compounds obtained: 138594346, 138594730, and 138594428 were observed with good binding affinities at binding energies of −15.0 kcal/mol, −14.6 kcal/mol, and −14.3 kcal/mol, respectively. In comparison, the reference molecule showed a binding energy of −13.8 kcal/mol, which substantiates the strong binding potential of the selected candidates. The best docking pose of each compound was re-docked into the target protein for validation purposes. This step points out the stability of the results obtained from screening and detailed views of protein-ligand interactions for each compound.

### ADMET analysis

The ADMET properties of the four candidate compounds (PubChem CIDs: 138594346, 138594730, 138594428, and a reference compound) were evaluated using the ADMETlab 2.0 web server. As shown in Supplementary S2 Table , all compounds exhibited low aqueous solubility (LogS ranging from −3.65 to −5.11) and moderate lipophilicity (LogP between 2.99 and 3.43), with high plasma protein binding percentages (>93%), suggesting limited free drug concentrations in plasma. Predicted human intestinal absorption (HIA) and Caco-2 permeability values indicated poor oral absorption, with very low bioavailability (F(20%) < 12%). Distribution profiles varied slightly, with compound 138594428 showing the highest blood-brain barrier (BBB) permeability (0.822). In terms of metabolism, all compounds displayed potential as substrates and inhibitors of key cytochrome P450 isoforms, notably CYP3A4 and CYP2C19, suggesting possible drug–drug interaction risks. Clearance and half-life predictions reflected moderate excretory profiles, with all compounds exhibiting short biological half-lives (t₁/₂ < 0.14 h). Toxicity evaluations indicated low AMES mutagenicity risks, while compound 138594428 showed the highest hERG inhibition (0.957) but the lowest drug-induced liver injury (DILI) probability (0.052). All compounds complied with Lipinski’s rule of five and were free of PAINS alerts, indicating good drug-likeness, though metabolic stability and bioavailability remain concerns.

### Molecular interactions

**Fig 4:**
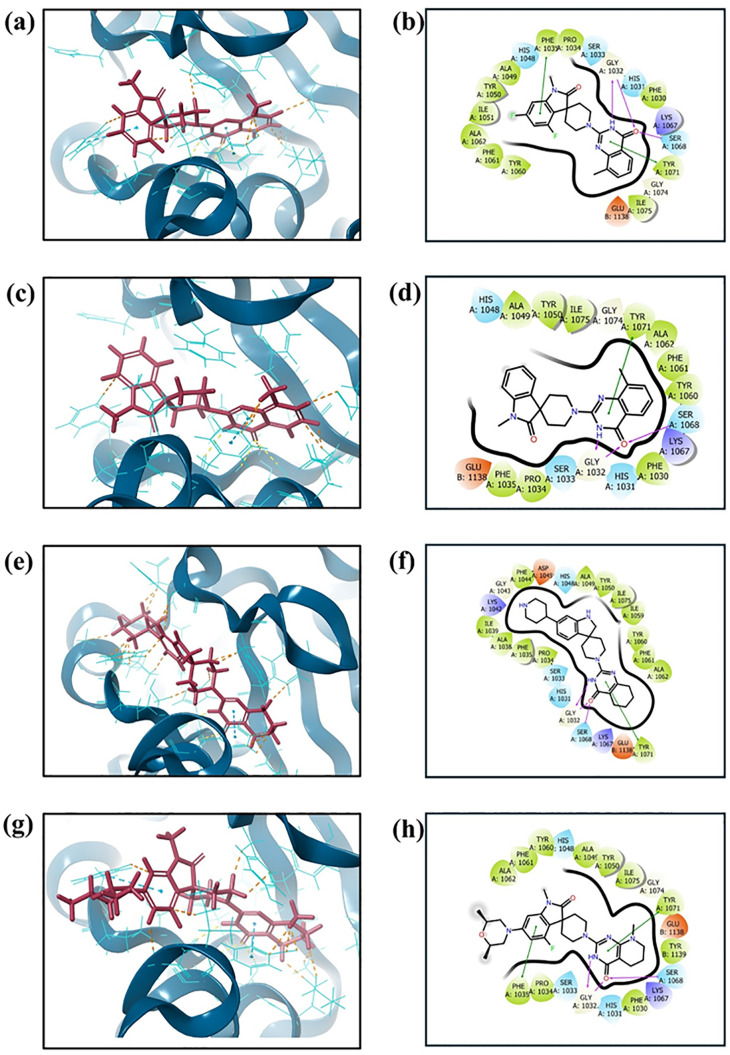
Protein-ligand binding modes and interaction maps for the top compounds and reference. Panels (a) 138594346, (c) 138594730, (e) 138594428, and (g) reference depict the 3D binding poses of ligands within the tankyrase active site, showing stabilizing interactions. Panels (b) 138594346, (d) 138594730, (f) 138594428, and (h) Reference present 2D interaction diagrams highlighting hydrogen bonds, hydrophobic contacts, and π-π stacking interactions with key residues.

Molecular interaction analysis was performed for the top candidates to show key interactions that give rise to high binding affinity (Fig 4). For Compound 138594346, hydrogen bonding was seen with Gly^1032^ (twice) and Ser^1068^. Hydrophobic interactions included Phe^1030^, Pro^1034^, Phe1035, Ala^1049^, Tyr^1050^, Ile^1051^, Tyr^1060^, Phe^1061^, Ala^1062^, Tyr^1071^, and Ile^1075^. Furthermore, π-π stacking interactions were seen with Phe^1035^ and Tyr^1071^. Similarly, compound 138594730 formed hydrogen bonds with Gly^1032^ twice and Ser^1068^. The hydrophobic interactions spanned residues such as Phe^1030^, Pro^1034^, Phe^1035^, Ala^1049^, Tyr^1050^, Tyr^1060^, Phe^1061^, Ala^1062^, Tyr^1071^, and Ile^1075^. Besides that, a π-π stacking interaction was noted with Tyr^1071^. Compound 138594428 formed hydrogen bonds with Gly^1032^ twice and Ser^1068^. It had hydrophobic interactions with Pro^1034^, Phe^1035^, Ala^1038^, Ile^1039^, Phe^1044^, Ala^1049^, Tyr^1050^, Ile^1059^, Tyr^1060^, Phe^1061^, Ala^1062^, Tyr^1071^, and Ile^1075^. π-π stacking was seen with Tyr^1071^. Similar binding patterns were also discussed in the previous report [42]. Hydrogen bonding with the reference compound was observed in two instances of Gly^1032^ and Ser^1068^. The following amino acid residues contributed to hydrophobic interactions: Phe^1030^, Pro^1034^, Phe^1035^, Ala^1049^, Tyr^1050^, Tyr^1060^, Phe^1061^, Ala^1062^, Tyr^1071^, Ile^1075^, and Tyr^1139^. π-π stacking was seen with Phe^1035^ and Tyr^1071^.

This indicates that Gly^1032^, Ser^1068^, and hydrophobic residues of the binding pocket, such as Phe^1035^ and Tyr^1071^, are essential in stabilizing ligand binding. The three top compounds exhibit comparable or better interactions than the reference compound. I have also studied the polar interaction patterns of the compounds with key pocket residues, including both hydrogen bond distances and angles. The relevant data is provided in Supplementary S1 Fig.

### Molecular dynamics simulation

#### Protein-ligand RMSD analysis.

**Fig 5:**
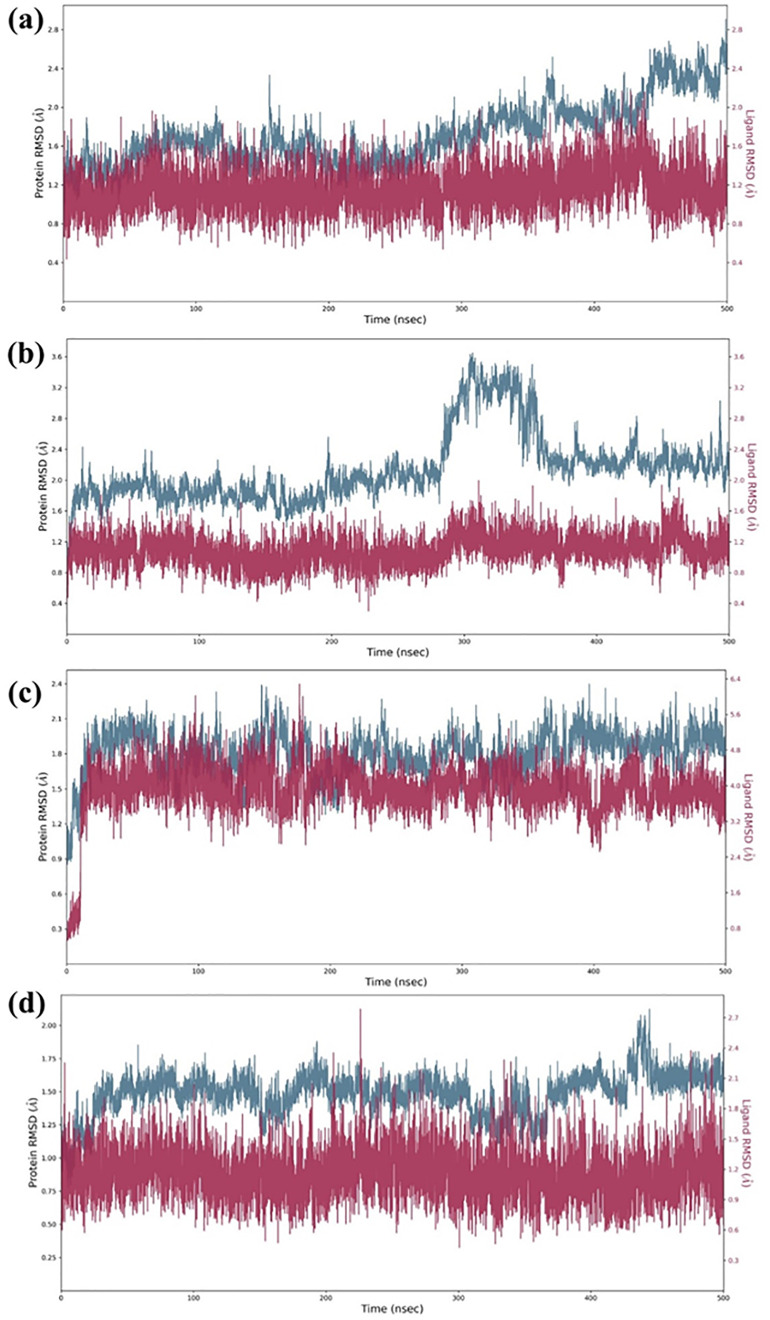
RMSD Analysis of the protein-ligand complexes: (a) 138594346, (b) 138594730, (c) 138594428, and (d) reference.

The RMSD profiles (Fig 5) illustrate the global structural stability of the protein-ligand complexes throughout the 500-ns MD simulation. Compound 138594346 showed consistently stable protein RMSD values (1.8 to 2.4 Å) in the last 100 ns, and its ligand RMSD was remarkably low (0.6–1.2 Å), reflecting high stability and snug fit in the binding site. These findings are very similar to the activity of the reference compound, which kept both protein and ligand RMSD values within comparably tight ranges (1.8–2.2 Å and 0.6–1.2 Å, respectively), again supporting the good dynamic profile of 138594346. Even though compound 138594730’s protein RMSD increased beyond 250 ns, to 3.2 Å, its ligand was stable with small fluctuations (0.6–1.0 Å) within the active site, indicating that the binding interaction was robust to protein conformational changes. Compound 138594428 demonstrated moderate protein RMSD (2.1–2.5 Å) and a relatively stable ligand RMSD, with a smaller increase to 2.4 Å by 300 ns, potentially indicative of adaptive motion rather than instability. Overall, these data point to 138594346 as by far the dynamically stable candidate, with 138594428 and 138594730 also promising by retaining ligand stability within the receptor’s binding pocket even with significant protein motion.

#### Protein and ligand RMSF analysis.

**Fig 6:**
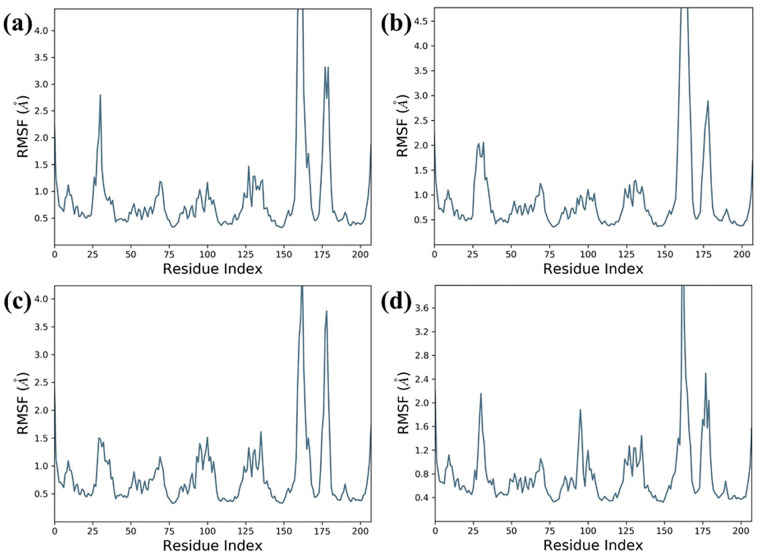
Analysis of Protein RMSF; (a) 138594346, (b) 138594730, (c) 138594428, and (d) reference.

**Fig 7:**
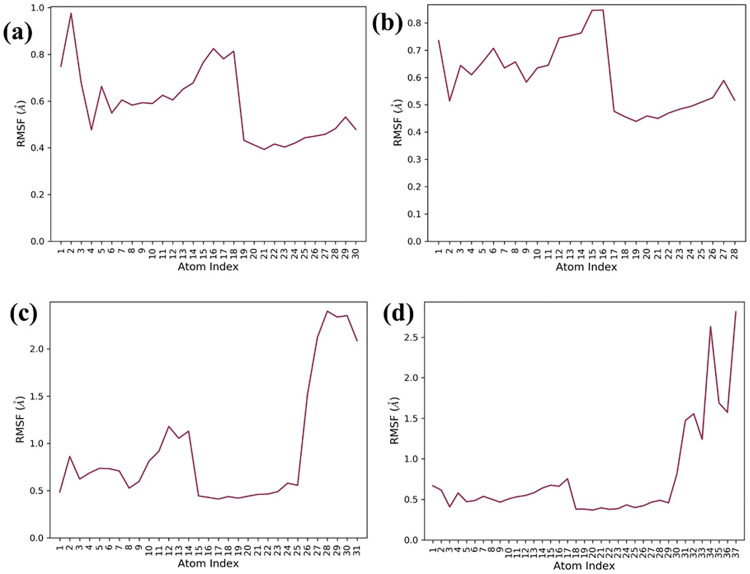
Analysis of Ligand RMSF; (a) 138594346, (b) 138594730, (c) 138594428, and (d) reference.

RMSF analysis (Fig 6) revealed expected flexibility in loop regions (residues 150–200) across all Tankyrase-2 complexes. Compound 138594346 showed moderate fluctuations (~3.5 Å at residue 175), indicating localized flexibility. 138594730 exhibited slightly higher peaks (~4.5 Å), consistent with its RMSD rise, while 138594428 showed moderate variation (~4.0 Å). The reference compound displayed the lowest fluctuations, with peaks around 3.6 Å, reflecting high structural stability. Ligand RMSF profiles (Fig 7) also reaffirmed these conclusions. 138594346 and 138594730 were stable with low atomic fluctuations (<0.8 Å), suggesting stable binding. While 138594428 demonstrated slightly elevated terminal flexibility (~2.5 Å), its core was stable. The reference ligand demonstrated lowest overall RMSF (<0.6 Å), reiterating strong and stable binding. In concert, 138594346 resembled very closely reference behavior, reaffirming its desirable dynamic profile.

### Enhanced stability of protein-ligand interactions Post-MD

**Fig 8:**
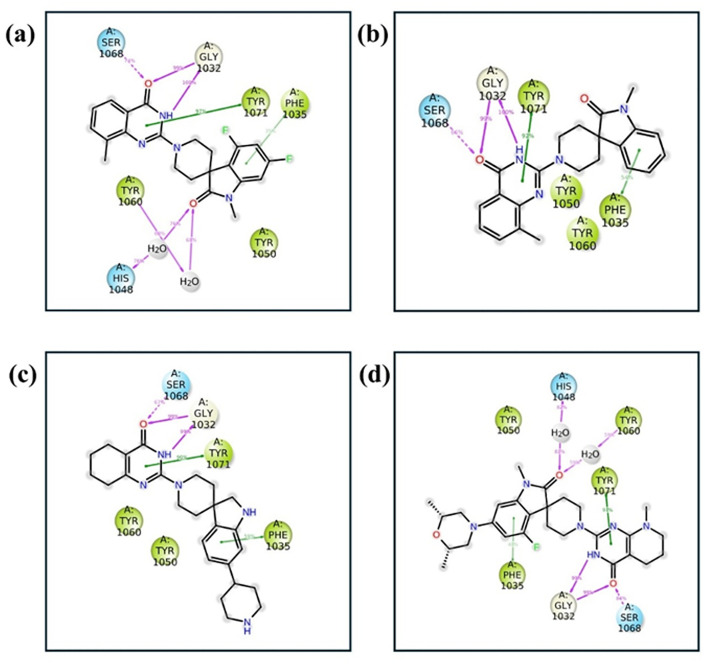
2D Interaction diagram of protein-ligand interactions obtained after MD simulation; (a) 138594346, (b) 138594730, (c) 138594428, and (d) reference.

**Fig 9:**
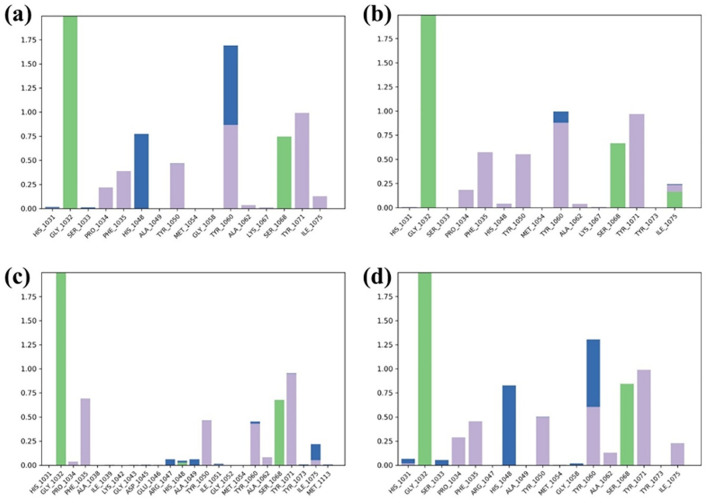
Histogram of protein-ligand interactions obtained after MD simulation; (a) 138594346, (b) 138594730, (c) 138594428, and (d) reference.

The post-MD study of interactions (Figs 8 and 9) also confirmed the retained and reinforced major docking interactions in all of the complexes, in particular the ones related to the binding defining residues of Tankyrase-2. For the compound 138594346, hydrogen bonding with Gly1032 (twice) and Ser1068 was maintained, whereas new contacts contributing to stability appeared with His1048 and Tyr1060. The hydrophobic contacts and the π–π stacking with Phe1035, Tyr1050, Tyr1060, and Tyr1071 remained in accordance with the docking, confirming its strong and stable binding.

Compound 138594730 preserved all key hydrogen bonds (Gly1032, Ser1068) and maintained hydrophobic and stacking contacts, mirroring the docking conformation with small deviation, suggesting good dynamic stability. Similarly, 138594428 maintained the pattern of hydrogen bonding and hydrophobic contacts, with preserved Phe1035 and Tyr1071 π–π stacking, verifying strong binding despite small ligand flexibility observed in RMSD. The reference substance also maintained its pattern of interaction, including the bonds with Gly1032, Ser1068, and His1048, and hydrophobic/π–π contacts with key aromatic residues, all of them favoring its stable conformation after simulation. Overall, the MD simulations validated and, in some cases, reinforced the interactions of docking, most importantly involving the core residues of Gly1032, Ser1068, His1048, and Tyr1060. These consistent interactions of all the ligands validate the result of docking and emphasize the dynamic stability and binding relevance of the top-ranked hits in the active site of Tankyrase-2.

### Machine learning-based prediction of biological activity

**Fig 10:**
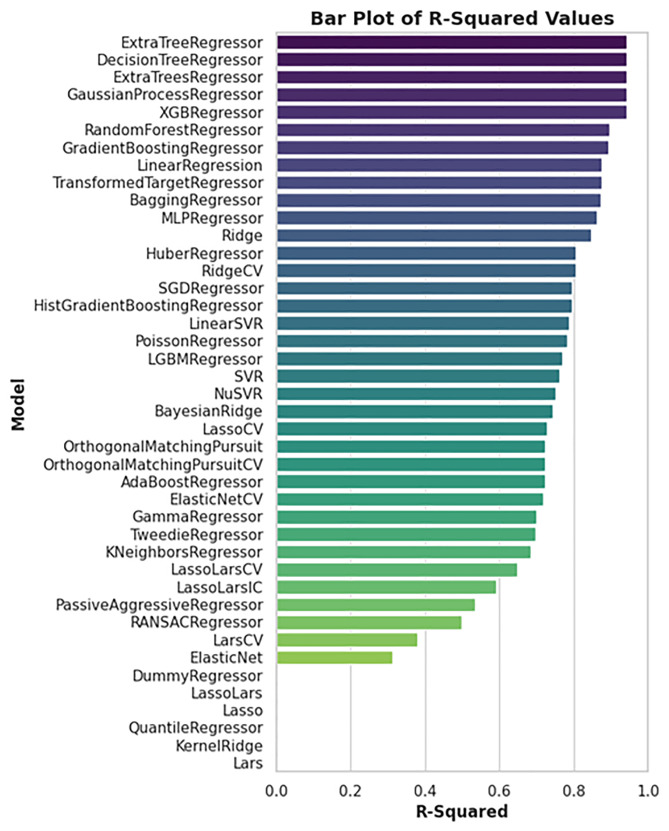
Comparison of R² values across 40 regression models. The ExtraTreeRegressor, XGBRegressor, and RandomForestRegressor models show superior performance with R² values above 0.9, indicating strong predictive accuracy.

**Fig 11:**
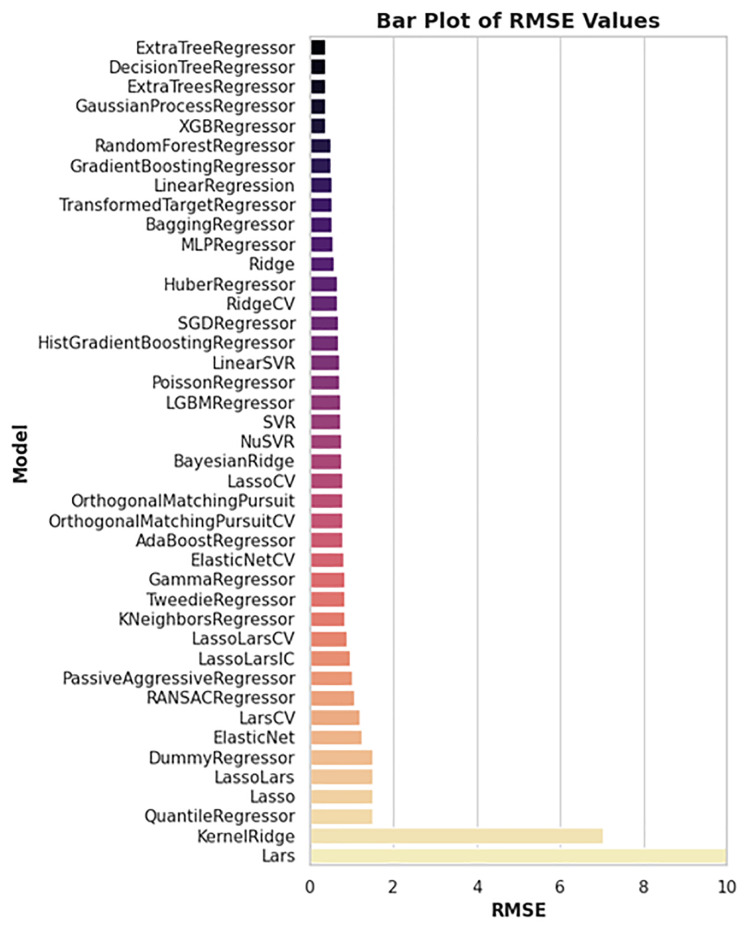
Comparison of RMSE values across 40 regression models. Models with the lowest RMSE—particularly ExtraTreeRegressor and XGBRegressor—demonstrate the best agreement between predicted and actual pIC₅₀ values.

**Fig 12:**
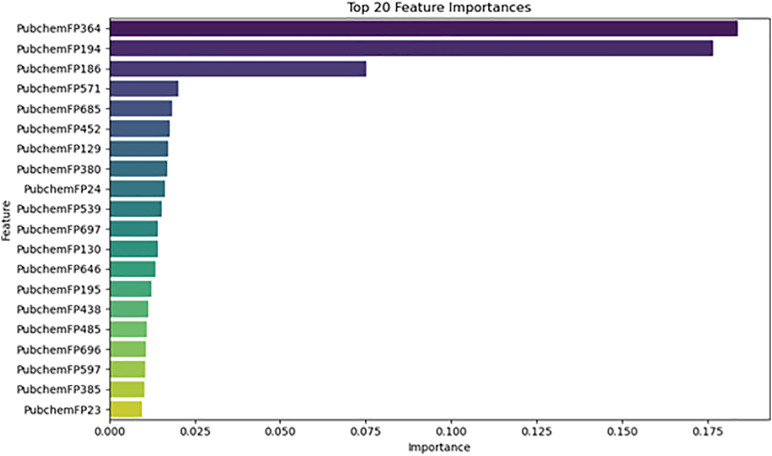
Top 20 most important molecular descriptors based on feature importance analysis. PubChem fingerprint features such as PubchemFP364, PubchemFP194, and PubchemFP186 contribute significantly to pIC₅₀ prediction.

A machine learning approach was applied to predict the biological activity (pIC₅₀) of newly identified Tankyrase inhibitors using a dataset of 236 known inhibitors from the ChEMBL database. Multiple regression models were evaluated, and their performance was compared based on R-squared (R²) and Root Mean Square Error (RMSE) metrics. As shown in (Fig 10), the top-performing models in terms of R² included ExtraTreeRegressor, DecisionTreeRegressor, and XGBRegressor, all achieving R² values near or above 0.95, indicating excellent predictive capability. The corresponding RMSE values (shown in Fig 11) confirm this trend, with these models yielding the lowest errors, suggesting minimal deviation from experimental values. Feature importance analysis, presented in (Fig 12), revealed that descriptors such as *PubchemFP364*, *PubchemFP194*, and *PubchemFP186* were among the most influential in predicting activity. These features likely represent key structural motifs contributing to inhibitory potential.

**Fig 13:**
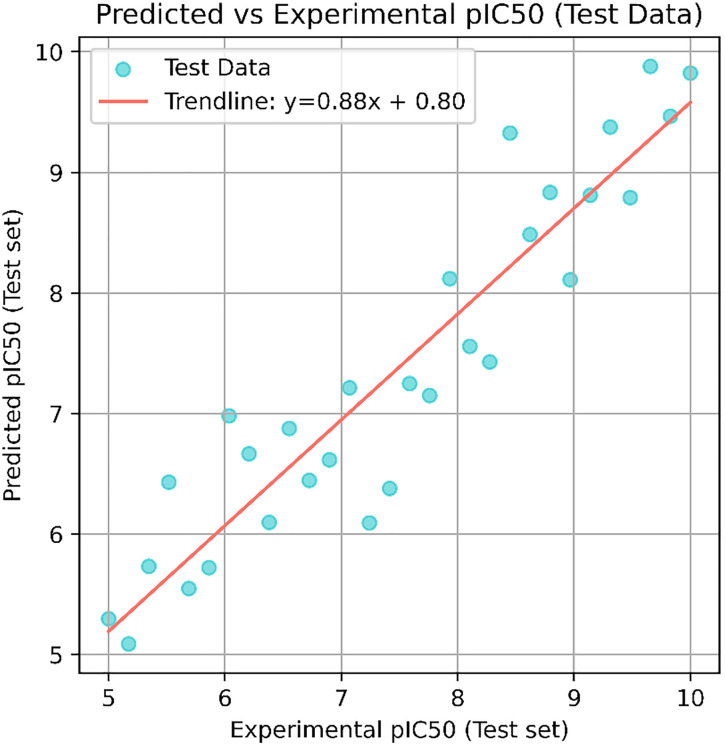
Predicted vs. experimental pIC_50_ values for the test set. A strong linear correlation illustrates the model’s ability to generalize well to unseen data.

**Fig 14:**
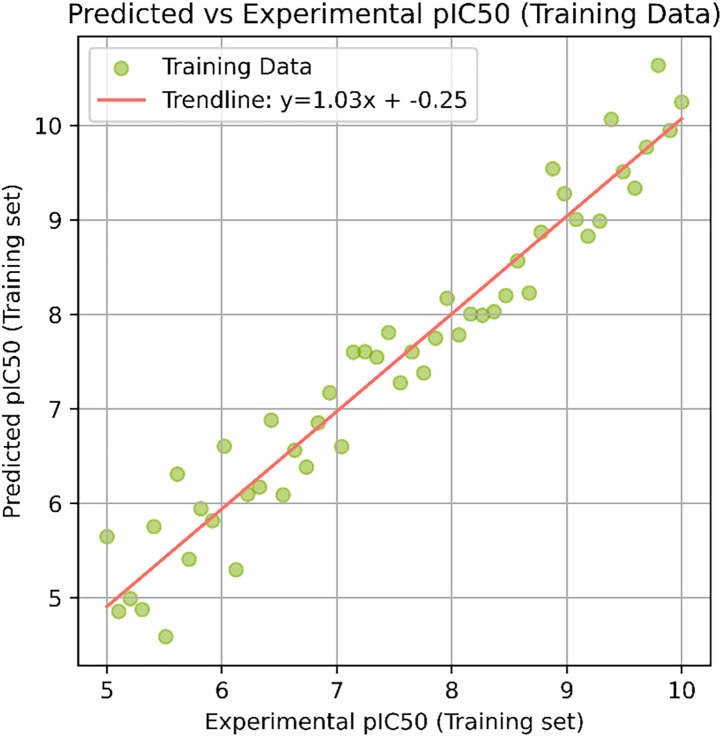
Predicted vs. experimental pIC_50_ values for the training set. The high correlation confirms accurate model fitting and low bias during training.

The final model demonstrated strong generalization ability, as reflected in the predicted vs. experimental pIC₅₀ plots for both training (Fig 13) and test sets (Fig 14). The training set showed a near-perfect fit (R² ≈ 0.95), while the test set also maintained high correlation (R² ≈ 0.88), indicating low risk of overfitting.

Predictions for the top three virtually screened compounds were as follows: 138594346 (7.70), 138594428 (7.41), and 138594730 (6.70), with the reference compound scoring the highest predicted pIC₅₀ of 7.71. Notably, compounds 138594346 and 138594428 exhibited a predicted activity closely approaching that of the reference, highlighting their potential as strong Tankyrase inhibitor candidates. To further validate the reliability of our ML predictions, I compared the predicted pIC₅₀ values with experimental data from the literature. Previous studies have reported Tankyrase inhibitors with pIC₅₀ values in the range of 7.0 to 8.0, indicating vigorous inhibitory activity against the target [14,43]. These studies support the accuracy of our ML model, as the predicted pIC₅₀ values for compounds 138594346 and 138594428 fall within the biologically relevant range, further reinforcing their potential for experimental validation and development.

### Scaffold similarity and novelty assessment

**Fig 15:**
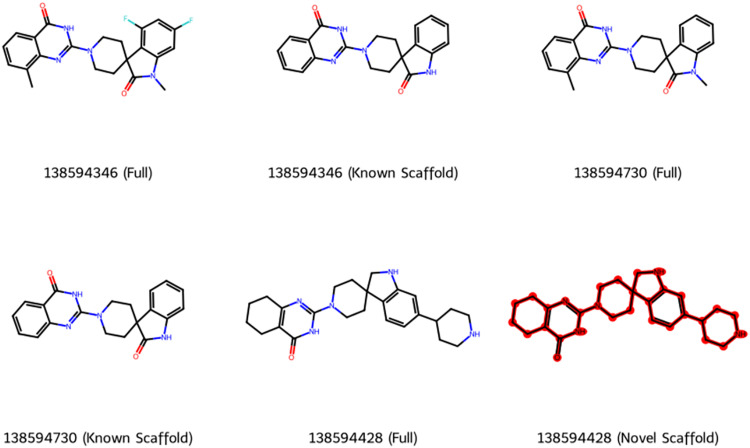
Scaffold comparison of the top three Tankyrase inhibitors. Full structures (left) and Bemis–Murcko scaffolds (right) are shown. 138594346 and 138594730 share known scaffolds, while 138594428 features a novel scaffold (red), absent from 236 ChEMBL-reported inhibitors.

To evaluate scaffold novelty, the top three candidate compounds were compared against 236 known Tankyrase inhibitors curated from the ChEMBL database using Bemis–Murcko scaffold decomposition. As shown in (Fig 15), each compound is represented in two forms: its full molecular structure, which includes all side chains and substituents, and its corresponding Bemis–Murcko scaffold, which captures the central ring system and linkers while removing peripheral groups. The decomposition analysis revealed that compound 138594428 contains a novel core scaffold (highlighted in red) that is absent from all known Tankyrase inhibitors in the reference dataset, suggesting a first-in-class chemotype. In contrast, compounds 138594346 and 138594730 exhibit known scaffolds, meaning their core frameworks are shared with previously reported Tankyrase inhibitors, indicating structural convergence with established Tankyrase-binding motifs. A reference compound bound to Tankyrase was identified from the crystal structure (PDB ID: 6KRO) and used as a template for similarity screening. Structural similarity searching was then performed against the PubChem database using its internal 80% similarity cutoff, which is based on PubChem’s own similarity metrics. This strategy was designed to explore structurally relevant regions of chemical space without overly constraining scaffold diversity. Within this filtered compound space, 138594346 and 138594730 were retrieved as scaffold-conserved hits, while 138594428, despite a moderate Tanimoto similarity of 0.714 to the reference ligand, introduces a Murcko scaffold not found among known Tankyrase inhibitors. This distinguishes 138594428 as a novel chemotype with the potential to expand the structural diversity of Tankyrase inhibition strategies and guide future optimization efforts.

## Discussion

Colorectal cancer (CRC) remains a significant global health concern, with rising incidence and mortality rates exacerbated by genetic mutations, particularly in the Wnt/β-catenin signaling pathway [44]. Tankyrase comes into play in this context as it plays a critical role in the regulation of this pathway; hence, tankyrase is also an interesting drug target in APC-mutated CRCs [43,45]. This work identified and validated novel tankyrase inhibitors using a computational approach, focusing on stability, interactions, and scaffold optimization.

Using virtual screening with an 80% similarity cutoff from the PubChem database, 533 compounds were retrieved; by considering Lipinski’s rule of five for drug-likeness, three potential candidates were selected: 138594346, 138594730, and 138594428, which show binding energies better than that of the reference compound, with values of −15.0 kcal/mol, −14.6 kcal/mol, and −14.3 kcal/mol, respectively (−13.8 kcal/mol) [46]. These results, therefore, point out that high binding affinity is obtained for the selected candidates, with strong molecular recognition suggested by the active site residues, in particular Gly^1032^ and Ser^1068^, as key contributors to hydrogen bonding.

DFT calculations revealed notable differences in electronic stability among the screened compounds. Compound 138594428 exhibited the largest HOMO-LUMO gap (4.979 eV), suggesting strong electronic stability and reduced reactivity—favorable traits for selective binding. Compound 138594346, which showed the most stable dynamics in MD simulations, had a slightly lower but still substantial gap of 4.473 eV, indicating a good balance between reactivity and stability. In contrast, the reference compound had the lowest gap (3.840 eV), reflecting higher reactivity and potentially less stable interactions. These results support the prioritization of both 138594428 and 138594346 for further analysis.

To validate the stability of these interactions under physiological conditions, the molecular dynamics for 500 ns were performed. Our analyses in RMSD showed that one compound had high stability, named 138594346. It had minor deviated values; the protein-ligand RMSD values lay between 1.8 and 2.4 angstroms and from 0.6 to 1.2 Å, respectively, comparable to the reference compound. On the other hand, 138594730 elicited considerable flexibility in a protein after a time period of 250 ns, while 138594428, after 300 ns, presents slight ligand rearrangements. The RMSF analysis after interacting proteins demonstrated peaks in highly flexible regions of the backbone between residues 150–180 and 175–200, without a doubt due to its already mentioned dynamic nature. Ligand’s RMSF data pointed out 138594346 as the most consistent structure, showing a small fraction of atomic fluctuation (less than 0.8Å), whereas 138594428 appeared flexible at the terminal end.

The post-MD interaction analysis showed that hydrogen bonds with Gly^1032^ and Ser^1068^, and additional interactions such as His^1048^ and Tyr^1060^, were maintained. These interactions further strengthened the stability observed during simulations [47]. Interestingly, hydrophobic interactions and π-π stacking with residues such as Phe1035 and Tyr1071 were retained in all top-ranked compounds, similar to the docking results [48]. The reference compound also presented similar stabilizing interactions, further validating the computational pipeline. These findings agree with recent studies highlighting the pivotal role of Gly1032 and hydrophobic residues for tankyrase-ligand binding.

Machine learning-based pIC₅₀ prediction offered critical insight into the potential inhibitory activity of the screened compounds. Among the candidates, 138594428 achieved a high predicted pIC₅₀ of 7.41, closely matching the reference compound (7.71), suggesting strong bioactivity. Notably, 138594346, which exhibited high structural stability in MD simulations, also showed a respectable predicted pIC₅₀ of 7.70, indicating it may also retain strong inhibitory potential. The model used for these predictions demonstrated high reliability, with strong R² scores and low RMSE on both training and test sets, reinforcing the validity of these results for prioritizing candidates like 138594428 and 138594346 for experimental follow-up.

Despite the robustness of this computational workflow, some limitations should be considered. Even though molecular dynamics simulations had been performed for 500 ns to ensure protein flexibility as well as validate the stability of protein-ligand links under dynamic environments, some segments of the workflow, such as the initial docking step, used a rigid receptor model, which might discount induced fit effects at the time of ligand binding. Additionally, the used force fields, though standard as well as well-established, may still pose intrinsic limitations to effectively model some of the non-covalent links or effects of polarization. The trained machine learning model, based on a dataset of 236 Tankyrase inhibitors, provided informative predictive insights. Yet, the relatively limited data size may constrain its applicability to more structurally diverse scaffolds. Lastly, although ADMET profiles had been predicted, future research should advantageously include synthetic accessibility scores as well as multi-objective optimization for enhanced hit prioritization. Experimental validation, however, remains essential; hence, subsequent in vitro and in vivo studies are necessary to confirm the predicted inhibitory activity and pharmacological relevance, as computational predictions do not always translate directly to biological efficacy.

## Conclusion

This study successfully applied an integrative computational strategy combining virtual screening, quantum chemical analysis, molecular docking, molecular dynamics (MD) simulations, and machine learning to identify and evaluate novel Tankyrase inhibitors for colorectal cancer (CRC) therapy. From an initial set of 533 compounds retrieved from the PubChem database, three top candidates, 138594346, 138594730, and 138594428, were identified based on their superior binding affinities compared to a known reference inhibitor.

To evaluate the electronic characteristics and chemical stability of the top compounds, DFT calculations were conducted. These revealed that 138594428 exhibited the largest HOMO-LUMO energy gap (4.979 eV), indicating high electronic stability and low polarizability, favorable for specific and stable binding interactions. In contrast, the reference compound displayed the lowest energy gap (3.840 eV), suggesting higher reactivity but potentially lower stability.

Subsequently, molecular docking studies revealed that the candidate compounds formed crucial interactions within the Tankyrase active site, particularly hydrogen bonds with Gly^1032^ and Ser^1068^, as well as hydrophobic and π–π stacking interactions. These findings were substantiated by molecular dynamics simulations, which assessed the dynamic behavior and stability of the complexes. Among the candidates, 138594346 showed exceptional conformational stability, with minimal deviations in RMSD and low fluctuations in RMSF analyses. Post-MD interaction profiling highlighted Gly^1032^, Ser^1068^, His^1048^, and Tyr^1060^ as critical residues contributing to complex stabilization.

Finally, a machine learning-based pIC₅₀ prediction model was employed to estimate the inhibitory potency of the selected compounds. Trained on 236 known Tankyrase inhibitors, the model predicted pIC₅₀ values of 7.70 (138594346), 6.70 (138594730), and 7.41 (138594428), with the reference compound scoring 7.71. These results further confirmed 138594346 and 138594428 as top candidates, showing both strong predicted activity and electronic stability, warranting further in vitro and in vivo investigation.

## Supporting information

S1 Table List of compounds obtained during virtual screening. (DOCX)

S2 Table ADMET analysis of top selected compounds. (DOCX)

S1 Fig Interaction patterns and hydrogen bond distances and angles between compounds and key pocket residues analysis for the top selected compounds along with the reference, (a) 138594346, (b) 138594730, (c) 138594428, and (d) reference. (DOCX)
