# Supplementary material for: Structural stability-guided scaffold hopping and computational modeling of tankyrase inhibitors targeting colorectal cancer
Source: PLoS One. 2025 Sep 19;20(9):e0332798. doi: 10.1371/journal.pone.0332798 (PMC12448342; doi:10.1371/journal.pone.0332798)
Supplement: S1 Table — (DOCX) [file pone.0332798.s001.docx]

| Compound | Energy |
| --- | --- |
| 138594346 | -15 |
| 138594730 | -14.6 |
| 138594428 | -14.3 |
| 155264207 | -14.3 |
| 146014936 | -14.1 |
| 138594627 | -13.9 |
| 138594197 | -13.9 |
| 138594262 | -13.8 |
| 138595067 | -13.8 |
| 138594231 | -13.8 |
| 155264271 | -13.8 |
| 138594194 | -13.8 |
| 138594178 | -13.8 |
| 138594645 | -13.7 |
| 138594614 | -13.7 |
| 146647612 | -13.7 |
| 142613964 | -13.7 |
| 138594862 | -13.7 |
| 161992595 | -13.7 |
| 138594227 | -13.7 |
| 138594272 | -13.6 |
| 138594472 | -13.6 |
| 152731079 | -13.6 |
| 138753113 | -13.6 |
| 155251293 | -13.6 |
| 138594563 | -13.6 |
| 147379908 | -13.6 |
| 138594744 | -13.6 |
| 138594171 | -13.6 |
| 138594510 | -13.6 |
| 138594155 | -13.5 |
| 138594836 | -13.5 |
| 138594292 | -13.5 |
| 160190029 | -13.5 |
| 161776875 | -13.5 |
| 138594198 | -13.4 |
| 138594172 | -13.4 |
| 138594959 | -13.4 |
| 138594652 | -13.4 |
| 138594202 | -13.4 |
| 138594628 | -13.4 |
| 138594570 | -13.4 |
| 146592301 | -13.4 |
| 138594208 | -13.4 |
| 138594179 | -13.3 |
| 137628286 | -13.3 |
| 138594790 | -13.3 |
| 138594309 | -13.3 |
| 142752300 | -13.3 |
| 138594559 | -13.3 |
| 138594271 | -13.3 |
| 138594503 | -13.3 |
| 138594322 | -13.3 |
| 138594176 | -13.3 |
| 138594632 | -13.3 |
| 138594507 | -13.3 |
| 138595134 | -13.3 |
| 138594671 | -13.2 |
| 138594497 | -13.2 |
| 155532717 | -13.2 |
| 138594964 | -13.2 |
| 155518184 | -13.2 |
| 138594558 | -13.2 |
| 138594747 | -13.2 |
| 138594393 | -13.2 |
| 138594353 | -13.2 |
| 153496987 | -13.2 |
| 138594238 | -13.2 |
| 155531429 | -13.2 |
| 138594350 | -13.2 |
| 138594692 | -13.1 |
| 138594474 | -13.1 |
| 138594182 | -13.1 |
| 138594185 | -13.1 |
| 138594434 | -13.1 |
| 139005963 | -13.1 |
| 141718236 | -13.1 |
| 138594230 | -13.1 |
| 138594696 | -13.1 |
| 146592318 | -13 |
| 138594470 | -13 |
| 156309801 | -13 |
| 138594404 | -13 |
| 138594400 | -13 |
| 138594287 | -13 |
| 142752297 | -13 |
| 138594438 | -12.9 |
| 138594169 | -12.9 |
| 138594414 | -12.9 |
| 138594791 | -12.9 |
| 138594505 | -12.9 |
| 146592306 | -12.9 |
| 138594439 | -12.9 |
| 155516254 | -12.9 |
| 153496988 | -12.8 |
| 138594256 | -12.8 |
| 138594642 | -12.8 |
| 138594395 | -12.8 |
| 137628287 | -12.8 |
| 146648282 | -12.8 |
| 138594233 | -12.8 |
| 155561438 | -12.7 |
| 155136634 | -12.7 |
| 138594599 | -12.7 |
| 138594242 | -12.7 |
| 138594655 | -12.7 |
| 138594157 | -12.7 |
| 138594423 | -12.7 |
| 155532179 | -12.6 |
| 138594167 | -12.6 |
| 138594602 | -12.6 |
| 135960332 | -12.6 |
| 136169834 | -12.6 |
| 136896656 | -12.5 |
| 138594546 | -12.5 |
| 138594952 | -12.5 |
| 138594687 | -12.5 |
| 136167394 | -12.5 |
| 138594814 | -12.5 |
| 155515281 | -12.5 |
| 136016064 | -12.5 |
| 138594910 | -12.5 |
| 138594381 | -12.5 |
| 156309804 | -12.5 |
| 138594209 | -12.5 |
| 136888376 | -12.5 |
| 138594917 | -12.4 |
| 151824520 | -12.4 |
| 138594572 | -12.4 |
| 141701195 | -12.4 |
| 138594485 | -12.4 |
| 138594426 | -12.4 |
| 138594906 | -12.4 |
| 160850281 | -12.4 |
| 138594813 | -12.4 |
| 138594399 | -12.4 |
| 138594213 | -12.4 |
| 136155961 | -12.4 |
| 137701512 | -12.4 |
| 155564702 | -12.4 |
| 162039498 | -12.4 |
| 138594564 | -12.4 |
| 155543325 | -12.4 |
| 142979279 | -12.4 |
| 138595170 | -12.4 |
| 138594596 | -12.3 |
| 138594606 | -12.3 |
| 138594235 | -12.3 |
| 112142749 | -12.3 |
| 138595172 | -12.3 |
| 146592302 | -12.3 |
| 146647615 | -12.3 |
| 155553178 | -12.3 |
| 138594151 | -12.3 |
| 138594336 | -12.3 |
| 155251253 | -12.2 |
| 138594582 | -12.2 |
| 138594170 | -12.2 |
| 145977639 | -12.2 |
| 136098470 | -12.2 |
| 136888331 | -12.2 |
| 136896657 | -12.2 |
| 138594731 | -12.2 |
| 138594852 | -12.2 |
| 136180579 | -12.1 |
| 136180415 | -12.1 |
| 136983632 | -12.1 |
| 135891740 | -12.1 |
| 136197630 | -12.1 |
| 138594321 | -12.1 |
| 137652348 | -12.1 |
| 138594806 | -12.1 |
| 138594181 | -12.1 |
| 138594199 | -12.1 |
| 138594837 | -12.1 |
| 138594506 | -12.1 |
| 136015816 | -12.1 |
| 155534087 | -12.1 |
| 138594491 | -12.1 |
| 138594639 | -12.1 |
| 138594430 | -12.1 |
| 136119818 | -12.1 |
| 159588025 | -12.1 |
| 138594407 | -12.1 |
| 138594354 | -12.1 |
| 138594429 | -12 |
| 138594160 | -12 |
| 138594574 | -12 |
| 138594168 | -12 |
| 156309800 | -12 |
| 138595179 | -12 |
| 138594640 | -12 |
| 138594206 | -12 |
| 55878201 | -12 |
| 138594921 | -12 |
| 112142750 | -12 |
| 138594499 | -12 |
| 138594300 | -12 |
| 112444847 | -11.9 |
| 137296597 | -11.9 |
| 135960333 | -11.9 |
| 136888361 | -11.9 |
| 122663698 | -11.9 |
| 138595106 | -11.9 |
| 157958690 | -11.9 |
| 112445634 | -11.9 |
| 138594291 | -11.9 |
| 146647614 | -11.9 |
| 156309802 | -11.9 |
| 138594409 | -11.8 |
| 138595128 | -11.8 |
| 112444848 | -11.8 |
| 138594310 | -11.8 |
| 138594483 | -11.8 |
| 28138482 | -11.8 |
| 112443797 | -11.8 |
| 138594249 | -11.8 |
| 138594748 | -11.8 |
| 138594284 | -11.8 |
| 136032145 | -11.8 |
| 136641626 | -11.7 |
| 136888364 | -11.7 |
| 138594844 | -11.7 |
| 137127789 | -11.7 |
| 137127790 | -11.7 |
| 138595137 | -11.7 |
| 141718242 | -11.7 |
| 138594487 | -11.7 |
| 136619597 | -11.7 |
| 112443798 | -11.7 |
| 137296672 | -11.7 |
| 136098469 | -11.7 |
| 136138833 | -11.7 |
| 136888360 | -11.7 |
| 153496974 | -11.6 |
| 138594896 | -11.6 |
| 138594539 | -11.6 |
| 132328906 | -11.6 |
| 153496986 | -11.6 |
| 76525137 | -11.6 |
| 136037392 | -11.6 |
| 112446226 | -11.5 |
| 135719667 | -11.5 |
| 148620900 | -11.5 |
| 155557028 | -11.5 |
| 112443662 | -11.5 |
| 155529963 | -11.5 |
| 146648623 | -11.5 |
| 136098483 | -11.4 |
| 136477766 | -11.4 |
| 145965857 | -11.4 |
| 136600825 | -11.4 |
| 112443661 | -11.4 |
| 136032164 | -11.4 |
| 136249382 | -11.4 |
| 137032330 | -11.4 |
| 142613969 | -11.4 |
| 138594618 | -11.3 |
| 146970192 | -11.3 |
| 138594411 | -11.3 |
| 145971867 | -11.3 |
| 145970539 | -11.2 |
| 135719668 | -11.2 |
| 110634532 | -11.2 |
| 112443895 | -11.2 |
| 66492809 | -11.2 |
| 28138481 | -11.2 |
| 136888365 | -11.2 |
| 136064273 | -11.2 |
| 155815642 | -11.2 |
| 136641616 | -11.2 |
| 16585307 | -11.2 |
| 112443875 | -11.2 |
| 43865437 | -11.2 |
| 112443876 | -11.2 |
| 112443896 | -11.2 |
| 112447764 | -11.1 |
| 120661371 | -11.1 |
| 76528762 | -11.1 |
| 137634187 | -11.1 |
| 136160321 | -11.1 |
| 136086638 | -11.1 |
| 112446225 | -11.1 |
| 76525183 | -11 |
| 120661372 | -11 |
| 136707096 | -11 |
| 136176178 | -11 |
| 138594765 | -11 |
| 159436745 | -11 |
| 136197711 | -11 |
| 135777201 | -11 |
| 138594413 | -11 |
| 142979232 | -11 |
| 155808772 | -11 |
| 136888368 | -11 |
| 135687231 | -11 |
| 112446795 | -10.9 |
| 136888377 | -10.9 |
| 136109694 | -10.9 |
| 165048875 | -10.9 |
| 112446796 | -10.9 |
| 112447051 | -10.9 |
| 112447059 | -10.9 |
| 146648425 | -10.9 |
| 164952389 | -10.9 |
| 112447052 | -10.9 |
| 112447060 | -10.8 |
| 136666557 | -10.8 |
| 112445940 | -10.8 |
| 138594201 | -10.8 |
| 136619603 | -10.8 |
| 164600699 | -10.8 |
| 111560217 | -10.8 |
| 135887580 | -10.8 |
| 139217054 | -10.8 |
| 111560363 | -10.8 |
| 136081748 | -10.8 |
| 137309573 | -10.8 |
| 136110382 | -10.8 |
| 111560364 | -10.8 |
| 144410235 | -10.7 |
| 145977802 | -10.7 |
| 136037391 | -10.7 |
| 136069617 | -10.7 |
| 155002615 | -10.7 |
| 136818331 | -10.7 |
| 144410236 | -10.7 |
| 110355064 | -10.7 |
| 136888362 | -10.7 |
| 89846290 | -10.7 |
| 66493465 | -10.7 |
| 155254058 | -10.7 |
| 148840656 | -10.7 |
| 136707095 | -10.7 |
| 141227493 | -10.7 |
| 159365779 | -10.6 |
| 110983658 | -10.6 |
| 112444614 | -10.6 |
| 159416126 | -10.6 |
| 43865465 | -10.6 |
| 112154765 | -10.6 |
| 136707092 | -10.6 |
| 136888367 | -10.6 |
| 146648525 | -10.6 |
| 136098498 | -10.6 |
| 119142619 | -10.6 |
| 146012842 | -10.6 |
| 136818330 | -10.6 |
| 136888332 | -10.6 |
| 136037388 | -10.5 |
| 137061040 | -10.5 |
| 136157756 | -10.5 |
| 138595111 | -10.5 |
| 138594402 | -10.5 |
| 66493458 | -10.5 |
| 135891741 | -10.5 |
| 119142702 | -10.5 |
| 59433640 | -10.4 |
| 136154478 | -10.4 |
| 135963184 | -10.4 |
| 112444296 | -10.4 |
| 157604926 | -10.4 |
| 112446116 | -10.4 |
| 136875109 | -10.4 |
| 112154764 | -10.4 |
| 58815740 | -10.4 |
| 161609583 | -10.4 |
| 136176218 | -10.4 |
| 135999685 | -10.4 |
| 167300173 | -10.4 |
| 136252522 | -10.3 |
| 136162926 | -10.3 |
| 158001311 | -10.3 |
| 112446802 | -10.3 |
| 76534590 | -10.3 |
| 119142703 | -10.3 |
| 136154446 | -10.3 |
| 111560218 | -10.3 |
| 111727939 | -10.2 |
| 145209962 | -10.2 |
| 157020102 | -10.2 |
| 136888363 | -10.2 |
| 136037401 | -10.2 |
| 112444918 | -10.2 |
| 112444917 | -10.2 |
| 146647617 | -10.2 |
| 135963183 | -10.2 |
| 136875110 | -10.2 |
| 111560443 | -10.1 |
| 126896798 | -10.1 |
| 57389785 | -10.1 |
| 112150397 | -10.1 |
| 111369191 | -10.1 |
| 137644906 | -10.1 |
| 112446970 | -10.1 |
| 155816426 | -10.1 |
| 111560444 | -10.1 |
| 136196449 | -10.1 |
| 145973922 | -10 |
| 112154775 | -10 |
| 170753875 | -10 |
| 136196454 | -10 |
| 70341043 | -10 |
| 112447677 | -10 |
| 120052780 | -10 |
| 112150401 | -10 |
| 112147155 | -10 |
| 112154774 | -10 |
| 112446617 | -10 |
| 112446618 | -10 |
| 57415220 | -10 |
| 136888330 | -9.9 |
| 138594435 | -9.9 |
| 112443491 | -9.9 |
| 136888375 | -9.9 |
| 112046045 | -9.9 |
| 112447678 | -9.9 |
| 28138480 | -9.9 |
| 16585306 | -9.9 |
| 136721443 | -9.9 |
| 111146891 | -9.9 |
| 136875107 | -9.9 |
| 134356312 | -9.9 |
| 119117446 | -9.8 |
| 111146890 | -9.8 |
| 112046044 | -9.8 |
| 110984874 | -9.8 |
| 120052781 | -9.8 |
| 135885274 | -9.8 |
| 73407652 | -9.8 |
| 137653139 | -9.8 |
| 137266944 | -9.8 |
| 112443492 | -9.8 |
| 119117447 | -9.8 |
| 17751555 | -9.8 |
| 89846293 | -9.7 |
| 57464219 | -9.7 |
| 137652191 | -9.7 |
| 146647649 | -9.7 |
| 135674550 | -9.7 |
| 124150558 | -9.7 |
| 138594289 | -9.7 |
| 68925583 | -9.7 |
| 156309805 | -9.7 |
| 143765921 | -9.6 |
| 112446450 | -9.6 |
| 136045675 | -9.6 |
| 170753509 | -9.6 |
| 110983660 | -9.6 |
| 111569507 | -9.6 |
| 112444678 | -9.5 |
| 142948372 | -9.5 |
| 112444677 | -9.5 |
| 17121973 | -9.5 |
| 112446099 | -9.5 |
| 136875105 | -9.5 |
| 112443678 | -9.5 |
| 138594728 | -9.5 |
| 169376401 | -9.5 |
| 158164239 | -9.5 |
| 112443677 | -9.5 |
| 112446100 | -9.4 |
| 28138837 | -9.4 |
| 138594822 | -9.4 |
| 136875111 | -9.4 |
| 135636712 | -9.4 |
| 138594635 | -9.3 |
| 136818332 | -9.3 |
| 135659213 | -9.3 |
| 146648472 | -9.3 |
| 16585305 | -9.3 |
| 109375918 | -9.3 |
| 138594641 | -9.3 |
| 135885273 | -9.3 |
| 136654003 | -9.3 |
| 126538813 | -9.3 |
| 136818329 | -9.3 |
| 28138477 | -9.3 |
| 170753885 | -9.3 |
| 158838134 | -9.3 |
| 126538503 | -9.2 |
| 28138479 | -9.2 |
| 142613971 | -9.2 |
| 164018398 | -9.2 |
| 109375919 | -9.2 |
| 136103078 | -9.1 |
| 154512141 | -9.1 |
| 137266943 | -9.1 |
| 56903809 | -9.1 |
| 95717815 | -9.1 |
| 87369871 | -9.1 |
| 138594394 | -9 |
| 112446449 | -9 |
| 136875104 | -8.9 |
| 136875108 | -8.9 |
| 138595048 | -8.9 |
| 171150067 | -8.9 |
| 69260556 | -8.9 |
| 95717814 | -8.9 |
| 132433297 | -8.9 |
| 135994198 | -8.8 |
| 68929541 | -8.8 |
| 28138836 | -8.8 |
| 28138478 | -8.8 |
| 28138484 | -8.7 |
| 28138485 | -8.6 |
| 28138483 | -8.6 |
| 59717521 | -8.6 |
| 16585308 | -8.6 |
| 28138486 | -8.5 |
| 16585309 | -8.5 |
| 157758071 | -8.5 |
| 97452605 | -8.5 |
| 110984873 | -8.5 |
| 55792 | -8.4 |
| 13147577 | -8.3 |
| 160393370 | -8.3 |
| 132433298 | -8.2 |
| 149116512 | -8.2 |
| 171150068 | -7.9 |
| 155811865 | -7.7 |
| 136875106 | -7.7 |
| 134923466 | -7.5 |
| 155807311 | -7.3 |
| 155805830 | -7 |
| 136641625 | -6.9 |
| 142979277 | -6.4 |
| 159075045 | -6.2 |
| 160356148 | -4 |

**Supplementary Table S1** – List of compounds obtained during virtual screening.
