# Supplementary material for: Structural stability-guided scaffold hopping and computational modeling of tankyrase inhibitors targeting colorectal cancer
Source: PLoS One. 2025 Sep 19;20(9):e0332798. doi: 10.1371/journal.pone.0332798 (PMC12448342; doi:10.1371/journal.pone.0332798)
Supplement: S2 Table — (DOCX) [file pone.0332798.s002.docx]

| **compounds** | **138594346** | **138594730** | **138594428** | **reference** |
| --- | --- | --- | --- | --- |
| smiles | Cc1cccc2c(=O)[nH]c(N3CCC4(CC3)C(=O)N(C)c3cc(F)cc(F)c34)nc12 | Cc1cccc2c(=O)[nH]c (N3CCC4(CC3)C(=O) N(C)c3ccccc34)nc12 | O=c1[nH]c(N2CCC3(CC2) CNc2cc(C4CCNCC4) ccc23)nc2c1CCCC2 | CC1CN(c2cc(F)c3c(c2)N(C) C(=O)C32CCN(c3nc(=O)c4c ([nH]3)N(C)CCC4)CC2)CC(C)O1 |
| LogS | -5.108 | -4.571 | -3.648 | -4.65 |
| LogD | 3.001 | 2.958 | 2.919 | 2.646 |
| LogP | 3.296 | 2.992 | 3.428 | 3.351 |
| Pgp-inh | 0.987 | 0.991 | 0.993 | 0.924 |
| Pgp-sub | 0.749 | 0.984 | 1 | 0.066 |
| HIA | 0.012 | 0.005 | 0.007 | 0.01 |
| F(20%) | 0.002 | 0.003 | 0.116 | 0.01 |
| F(30%) | 0.016 | 0.003 | 0.64 | 0.089 |
| Caco-2 | -4.959 | -4.959 | -5.581 | -4.966 |
| MDCK | 2.35E-05 | 2.26E-05 | 2.68E-06 | 1.44E-05 |
| BBB | 0.141 | 0.568 | 0.822 | 0.158 |
| PPB | 97.39% | 96.97% | 93.33% | 94.84% |
| VDss | 0.919 | 0.768 | 2.471 | 1.401 |
| Fu | 2.03% | 1.61% | 4.70% | 4.77% |
| CYP1A2-inh | 0.408 | 0.508 | 0.237 | 0.075 |
| CYP1A2-sub | 0.947 | 0.941 | 0.829 | 0.493 |
| CYP2C19-inh | 0.821 | 0.847 | 0.188 | 0.581 |
| CYP2C19-sub | 0.813 | 0.852 | 0.075 | 0.85 |
| CYP2C9-inh | 0.816 | 0.799 | 0.078 | 0.351 |
| CYP2C9-sub | 0.114 | 0.117 | 0.02 | 0.062 |
| CYP2D6-inh | 0.448 | 0.51 | 0.875 | 0.268 |
| CYP2D6-sub | 0.141 | 0.159 | 0.838 | 0.458 |
| CYP3A4-inh | 0.549 | 0.446 | 0.607 | 0.427 |
| CYP3A4-sub | 0.797 | 0.91 | 0.575 | 0.921 |
| CL | 4.447 | 4.424 | 4.542 | 3.539 |
| T12 | 0.131 | 0.138 | 0.047 | 0.108 |
| hERG | 0.107 | 0.337 | 0.957 | 0.211 |
| H-HT | 0.71 | 0.465 | 0.765 | 0.974 |
| DILI | 0.949 | 0.957 | 0.052 | 0.947 |
| Ames | 0.893 | 0.744 | 0.598 | 0.727 |
| ROA | 0.71 | 0.909 | 0.954 | 0.979 |
| FDAMDD | 0.853 | 0.818 | 0.967 | 0.917 |
| SkinSen | 0.092 | 0.053 | 0.085 | 0.082 |
| Carcinogenicity | 0.796 | 0.927 | 0.613 | 0.952 |
| EC | 0.003 | 0.003 | 0.003 | 0.003 |
| EI | 0.01 | 0.01 | 0.007 | 0.006 |
| Respiratory | 0.094 | 0.034 | 0.942 | 0.253 |
| BCF | 2.012 | 1.56 | 1.339 | 1.712 |
| IGC50 | 3.094 | 2.899 | 2.789 | 3.233 |
| LC50 | 4.555 | 4.543 | 4.079 | 3.832 |
| LC50DM | 6.523 | 5.797 | 6.251 | 5.929 |
| NR-AR | 0.019 | 0.027 | 0.016 | 0.041 |
| NR-AR-LBD | 0.144 | 0.305 | 0.004 | 0.276 |
| NR-AhR | 0.455 | 0.519 | 0.344 | 0.51 |
| NR-Aromatase | 0.066 | 0.077 | 0.521 | 0.289 |
| NR-ER | 0.158 | 0.399 | 0.213 | 0.468 |
| NR-ER-LBD | 0.008 | 0.068 | 0.03 | 0.446 |
| NR-PPAR-gamma | 0.898 | 0.662 | 0.014 | 0.94 |
| SR-ARE | 0.791 | 0.793 | 0.652 | 0.822 |
| SR-ATAD5 | 0.008 | 0.07 | 0.012 | 0.083 |
| SR-HSE | 0.011 | 0.009 | 0.026 | 0.019 |
| SR-MMP | 0.403 | 0.492 | 0.155 | 0.591 |
| SR-p53 | 0.918 | 0.922 | 0.637 | 0.966 |
| MW | 410.16 | 374.17 | 419.27 | 510.28 |
| Vol | 396.26 | 384.125 | 437.573 | 504.173 |
| Dense | 1.035 | 0.974 | 0.958 | 1.012 |
| nHA | 6 | 6 | 6 | 9 |
| nHD | 1 | 1 | 3 | 1 |
| TPSA | 69.3 | 69.3 | 73.05 | 85.01 |
| nRot | 1 | 1 | 2 | 2 |
| nRing | 5 | 5 | 6 | 6 |
| MaxRing | 10 | 10 | 10 | 10 |
| nHet | 8 | 6 | 6 | 10 |
| fChar | 0 | 0 | 0 | 0 |
| nRig | 29 | 29 | 34 | 35 |
| Flex | 0.034 | 0.034 | 0.059 | 0.057 |
| nStereo | 0 | 0 | 0 | 2 |
| NonBiodegradable | 1 | 0 | 0 | 2 |
| NonGenotoxic_Carcinogenicity | 0 | 0 | 0 | 1 |
| SureChEMBL | 0 | 0 | 0 | 0 |
| LD50_oral | 0 | 0 | 0 | 0 |
| Skin_Sensitization | 1 | 1 | 2 | 1 |
| Acute_Aquatic_Toxicity | 1 | 0 | 0 | 1 |
| Toxicophores | 1 | 1 | 1 | 1 |
| Genotoxic_Carcinogenicity_Mutagenicity | 1 | 1 | 1 | 1 |
| QED | 0.67 | 0.711 | 0.698 | 0.664 |
| Synth | 3.433 | 3.102 | 3.641 | 4.639 |
| Fsp3 | 0.318 | 0.318 | 0.6 | 0.593 |
| MCE-18 | 100.345 | 93.655 | 112.5 | 164.093 |
| Natural Product-likeness | -1.073 | -0.795 | -0.446 | -0.815 |
| Alarm_NMR | 0 | 0 | 0 | 0 |
| BMS | 0 | 0 | 0 | 0 |
| Chelating | 0 | 0 | 0 | 0 |
| PAINS | 0 | 0 | 0 | 0 |
| Lipinski | Accepted | Accepted | Accepted | Accepted |
| Pfizer | Rejected | Accepted | Rejected | Accepted |
| GSK | Rejected | Accepted | Rejected | Rejected |
| GoldenTriangle | Accepted | Accepted | Accepted | Rejected |

**Supplementary Table S2 –** ADMET analysis of top selected compounds.
