# Supplementary material for: Structural stability-guided scaffold hopping and computational modeling of tankyrase inhibitors targeting colorectal cancer
Source: PLoS One. 2025 Sep 19;20(9):e0332798. doi: 10.1371/journal.pone.0332798 (PMC12448342; doi:10.1371/journal.pone.0332798)
Supplement: S1 Fig — (DOCX) [file pone.0332798.s003.docx]

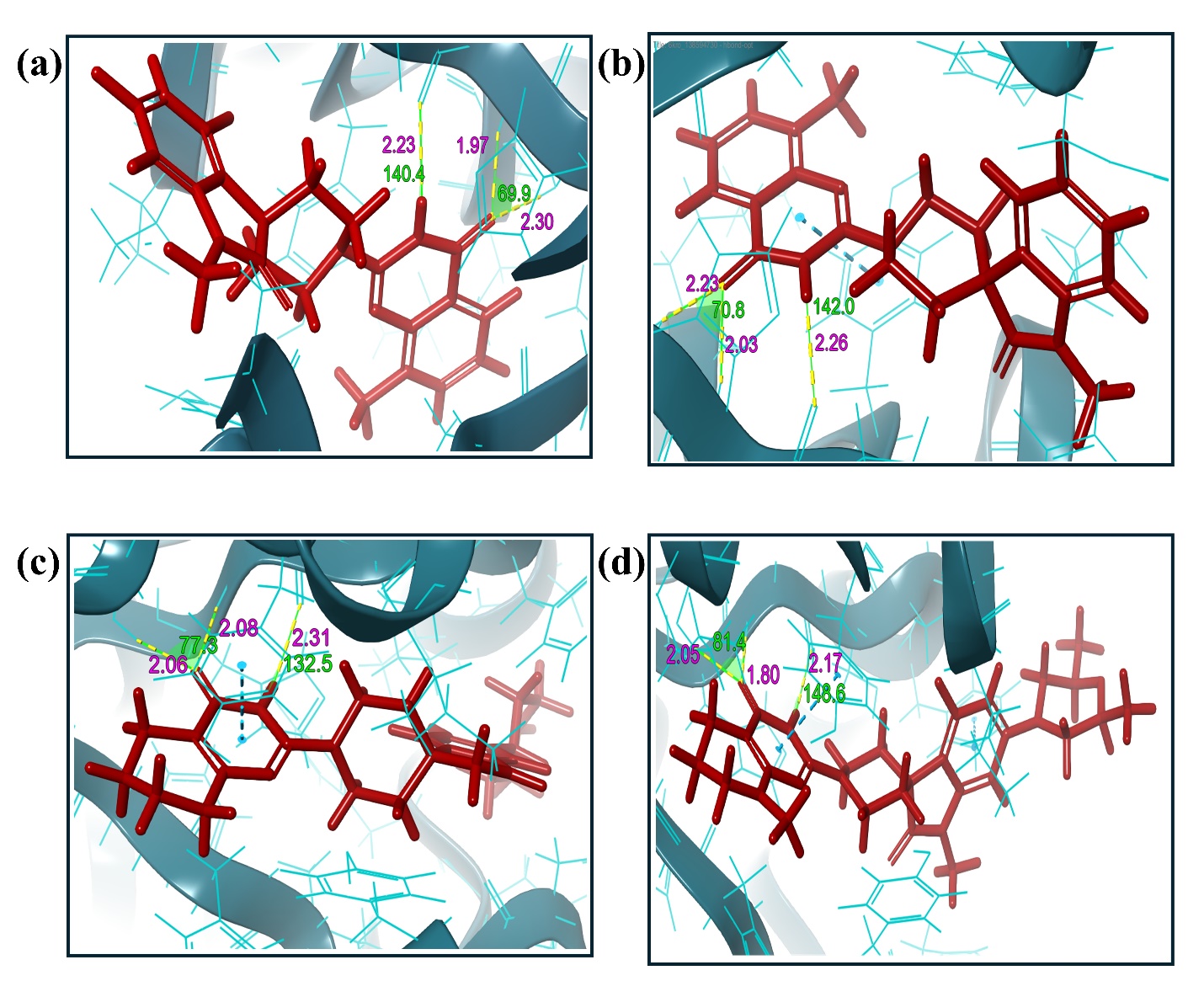
**Supplementary Figure S1 -** Interaction patterns and hydrogen bond distances and angles between compounds and key pocket residues analysis for the top selected compounds along with the reference, (a) 138594346, (b) 138594730, (c) 138594428, and (d) reference.
