# Supplementary material for: Structural stability-guided scaffold hopping and computational modeling of tankyrase inhibitors targeting colorectal cancer
Source: PLoS One. 2025 Sep 19;20(9):e0332798. doi: 10.1371/journal.pone.0332798 (PMC12448342; doi:10.1371/journal.pone.0332798)
Supplement: Attachment — Submitted filename: Response to reviewers.docx [file pone.0332798.s005.docx]

**Response Letter**

**Reviewer 1:**

**Comment 1 -** Lack of Experimental Validation: While the computational results are promising, the manuscript would benefit from a clearer discussion of the next steps for in vitro/in vivo validation, especially given that predictions can sometimes fail to translate biologically.

**Response –** Dear Sir, I appreciate your valuable suggestion. To address this point, I have expanded the Discussion section to include a specific paragraph highlighting the need for experimental validation.

**Comment 2** - It is not entirely clear how novel the top three compounds are in terms of chemical scaffolds compared to known Tankyrase inhibitors. A comparison with structurally similar known inhibitors would strengthen the novelty claim.

**Response –** Thank you for the valuable comment. A scaffold-level comparison has been added to clarify the novelty of the top compounds.

**Comment 3 -** More details are needed on how the ML model was selected and validated, particularly the choice of descriptor types and hyperparameters for the final model.

**Response -** I thank you for the suggestion. The Methods section has been revised to include details on descriptor types, model selection, hyperparameter tuning, and validation strategy.

**Comment 4 -** Some sections, particularly the MD simulation and interaction discussion, are repetitive and could be condensed without loss of clarity.

**Response –** I thank you for the observation. The MD simulation and interaction sections have been revised to remove redundancy and improve clarity.

**Reviewer 2:**

**Comment -1** Excellent integration of multiple computational techniques: DFT + MD + ML + Docking. Logical flow from database screening to MD validation and predictive modeling. But you need to add experimental or literature-based activity values for validation of the ML predictions. Include visual summaries or flowcharts for the workflow (database → docking → DFT → MD → ML).

Discuss limitations of the computational approach (e.g., force field limitations, ML data size, docking rigidity). Consider using ADMET profiling or synthetic accessibility scores to prioritize the hits.

**Response –** Dear Sir, I thank you for the positive feedback and constructive suggestions. I have added literature-based activity values to support ML predictions and included a workflow diagram summarizing the computational pipeline. Limitations of the approach and comments on ADMET profiling and synthetic accessibility have been addressed in the revised Discussion section.

**Reviewer 3:**

*The authors of the provided manuscript explored the potential anti-cancer activity of PubChem-deposited small molecule inhibitors of tankyrase biotarget. Comprehensive in silico study was performed over multi-stage screening stage to identify the potential hits. The manuscript is relevant in the field of drug discovery. Comments are to be addressed as follows:

**Response -** I would like to thank you for your thoughtful comments and constructive feedback. I appreciate the time and effort taken to review my manuscript, and I am grateful for your recognition of the relevance of my work in the field of drug discovery. I have carefully addressed the comments provided to improve the manuscript further.

**Comment 1 -** In the molecular modelling simulation section, brief description regarding the tankyrase’s topology, binding site description, and key binding residues being highlighted important within the current literature should be provided.

**Response -** I have added a brief description of tankyrase’s domain architecture, binding site features, and key residues at the beginning of the Molecular Dynamics Simulation section to clarify the structural basis for ligand interaction.

**Comment 2 -** Further, the compounds’ polar interaction patterns with key pocket residues should be annotated in terms of both the bond distances and angles. Hydrogen bonding should be presented within hydrogen bond distances as well as bond angles since hydrogen bond depend on both. Authors should mention the Hydrogen bond angles as well as their distances, since the strength of hydrogen bonding is based on both parameters in a way to ensure the adequacy of optimum hydrogen bonding.

**Response -** Thank you for your suggestion. I have included both hydrogen bond distances and angles in the revised manuscript, with the detailed information provided in Supplementary Figure 1.

**Comment 3** - The authors provided Free binding energy calculations out of the MD simulation trajectories. It is advised to provide the dissected energy terms of these total energies (ΔGbind Lipo, ΔGbind Solv GB, ΔGbind vDW, ΔGbind Coulomb, and ΔGbind Ligand strain) to further account for simulated solvation entropy and evaluate the nature of interaction (dominant energy potential) based on these different energy terms. This would guide further hit to lead and lead optimization steps.

**Response –** I sincerely thank you for this insightful suggestion. I fully agree that decomposing the binding energy would offer essential insights; however, I was unable to perform this analysis due to the lack of access to the required licensed software. I kindly request your understanding in this regard.

**Comment 4 -** Based on the study results, what are the take-away messages. Authors are advised to highlight the future suggested structural modifications that would improve the hits’ predicted activities based on the computational findings. These insights would be beneficial for guiding future lead optimization and development.

**Response -** I thank you for the valuable suggestion. A summary of key interaction features and proposed structural modifications has been added to help guide future lead optimization and development.
