# Supplementary material for: Structural stability-guided scaffold hopping and computational modeling of tankyrase inhibitors targeting colorectal cancer
Source: PLoS One. 2025 Sep 19;20(9):e0332798. doi: 10.1371/journal.pone.0332798 (PMC12448342; doi:10.1371/journal.pone.0332798)
Supplement: Peer review history

# Peer review: Decision Letter 0

**DOI:** 10.1371/journal.pone.0332798.r001

**Authors:** Ahmed Al-Karmalawy

21 Jul 2025

Dear Dr. Alshahrani,

Thank you for submitting your manuscript to PLOS ONE. After careful consideration, we feel that it has merit but does not fully meet PLOS ONE’s publication criteria as it currently stands. Therefore, we invite you to submit a revised version of the manuscript that addresses the points raised during the review process.

Please submit your revised manuscript by Sep 04 2025 11:59PM. If you will need more time than this to complete your revisions, please reply to this message or contact the journal office at plosone@plos.org . When you're ready to submit your revision, log on to https://www.editorialmanager.com/pone/ and select the 'Submissions Needing Revision' folder to locate your manuscript file.

- A rebuttal letter that responds to each point raised by the academic editor and reviewer(s). You should upload this letter as a separate file labeled 'Response to Reviewers'.
- A marked-up copy of your manuscript that highlights changes made to the original version. You should upload this as a separate file labeled 'Revised Manuscript with Track Changes'.
- An unmarked version of your revised paper without tracked changes. You should upload this as a separate file labeled 'Manuscript'.

If applicable, we recommend that you deposit your laboratory protocols in protocols.io to enhance the reproducibility of your results. Protocols.io assigns your protocol its own identifier (DOI) so that it can be cited independently in the future. For instructions see: https://journals.plos.org/plosone/s/submission-guidelines#loc-laboratory-protocols . Additionally, PLOS ONE offers an option for publishing peer-reviewed Lab Protocol articles, which describe protocols hosted on protocols.io. Read more information on sharing protocols at https://plos.org/protocols?utm_medium=editorial-email&utm_source=authorletters&utm_campaign=protocols .

We look forward to receiving your revised manuscript.

Kind regards,

Ahmed A. Al-Karmalawy, PhD

Academic Editor

PLOS ONE

Journal Requirements:

When submitting your revision, we need you to address these additional requirements.

1. Please ensure that your manuscript meets PLOS ONE's style requirements, including those for file naming. The PLOS ONE style templates can be found at

https://journals.plos.org/plosone/s/file?id=wjVg/PLOSOne_formatting_sample_main_body.pdf and

https://journals.plos.org/plosone/s/file?id=ba62/PLOSOne_formatting_sample_title_authors_affiliations.pdf

2. Please note that PLOS One has specific guidelines on code sharing for submissions in which author-generated code underpins the findings in the manuscript. In these cases, we expect all author-generated code to be made available without restrictions upon publication of the work. Please review our guidelines at https://journals.plos.org/plosone/s/materials-and-software-sharing#loc-sharing-code and ensure that your code is shared in a way that follows best practice and facilitates reproducibility and reuse.

3. Please include captions for your Supporting Information files at the end of your manuscript, and update any in-text citations to match accordingly. Please see our Supporting Information guidelines for more information: http://journals.plos.org/plosone/s/supporting-information.

4. If the reviewer comments include a recommendation to cite specific previously published works, please review and evaluate these publications to determine whether they are relevant and should be cited. There is no requirement to cite these works unless the editor has indicated otherwise. 

5. Please review your reference list to ensure that it is complete and correct. If you have cited papers that have been retracted, please include the rationale for doing so in the manuscript text, or remove these references and replace them with relevant current references. Any changes to the reference list should be mentioned in the rebuttal letter that accompanies your revised manuscript. If you need to cite a retracted article, indicate the article’s retracted status in the References list and also include a citation and full reference for the retraction notice.

[Note: HTML markup is below. Please do not edit.]

Reviewers' comments:

Reviewer's Responses to Questions

**Comments to the Author**

1. Is the manuscript technically sound, and do the data support the conclusions?

Reviewer #1: Partly

Reviewer #2: Yes

Reviewer #3: Yes

**********

2. Has the statistical analysis been performed appropriately and rigorously?

Reviewer #1: N/A

Reviewer #2: Yes

Reviewer #3: N/A

**********

3. Have the authors made all data underlying the findings in their manuscript fully available?

The PLOS Data policy

Reviewer #1: Yes

Reviewer #2: Yes

Reviewer #3: Yes

**********

4. Is the manuscript presented in an intelligible fashion and written in standard English?

Reviewer #1: Yes

Reviewer #2: No

Reviewer #3: Yes

**********

Reviewer #1: 1.Lack of Experimental Validation: While the computational results are promising, the manuscript would benefit from a clearer discussion of the next steps for in vitro/in vivo validation, especially given that predictions can sometimes fail to translate biologically.

2.It is not entirely clear how novel the top three compounds are in terms of chemical scaffolds compared to known Tankyrase inhibitors. A comparison with structurally similar known inhibitors would strengthen the novelty claim.

3.More details are needed on how the ML model was selected and validated, particularly the choice of descriptor types and hyperparameters for the final model.

4.Some sections, particularly the MD simulation and interaction discussion, are repetitive and could be condensed without loss of clarity.

Reviewer #2: Excellent integration of multiple computational techniques: DFT + MD + ML + Docking.

Logical flow from database screening to MD validation and predictive modeling.

But you need to add experimental or literature-based activity values for validation of the ML predictions.

Include visual summaries or flowcharts for the workflow (database → docking → DFT → MD → ML).

Discuss limitations of the computational approach (e.g., force field limitations, ML data size, docking rigidity).

Consider using ADMET profiling or synthetic accessibility scores to prioritize the hits.

Reviewer #3: The authors of the provided manuscript explored the potential anti-cancer activity of PubChem-deposited small molecule inhibitors of tankyrase biotarget. Comprehensive in silico study was performed over multi-stage screening stage to identify the potential hits. The manuscript is relevant in the field of drug discovery. Comments are to be addressed as follows:

1. In the molecular modelling simulation section, brief description regarding the tankyrase’s topology, binding site description, and key binding residues being highlighted important within the current literature should be provided.

2. Further, the compounds’ polar interaction patterns with key pocket residues should be annotated in terms of both the bond distances and angles. Hydrogen bonding should be presented within hydrogen bond distances as well as bond angles since hydrogen bond depend on both. Authors should mention the Hydrogen bond angles as well as their distances, since the strength of hydrogen bonding is based on both parameters in a way to ensure the adequacy of optimum hydrogen bonding.

3. The authors provided Free binding energy calculations out of the MD simulation trajectories. It is advised to provide the dissected energy terms of these total energies (ΔGbind Lipo, ΔGbind Solv GB, ΔGbind vDW, ΔGbind Coulomb, and ΔGbind Ligand strain) to further account for simulated solvation entropy and evaluate the nature of interaction (dominant energy potential) based on these different energy terms. This would guide further hit to lead and lead optimization steps.

4. Based on the study results, what are the take-away messages. Authors are advised to highlight the future suggested structural modifications that would improve the hits’ predicted activities based on the computational findings. These insights would be beneficial for guiding future lead optimization and development.

**********

what does this mean? ). If published, this will include your full peer review and any attached files.

If you choose “no”, your identity will remain anonymous but your review may still be made public.

**Do you want your identity to be public for this peer review?** For information about this choice, including consent withdrawal, please see our Privacy Policy

Reviewer #1: No

Reviewer #2: **Yes**

Reviewer #3: **Yes**

**********

[NOTE: If reviewer comments were submitted as an attachment file, they will be attached to this email and accessible via the submission site. Please log into your account, locate the manuscript record, and check for the action link "View Attachments". If this link does not appear, there are no attachment files.]

While revising your submission, please upload your figure files to the Preflight Analysis and Conversion Engine (PACE) digital diagnostic tool, https://pacev2.apexcovantage.com/ . PACE helps ensure that figures meet PLOS requirements. To use PACE, you must first register as a user. Registration is free. Then, login and navigate to the UPLOAD tab, where you will find detailed instructions on how to use the tool. If you encounter any issues or have any questions when using PACE, please email PLOS at figures@plos.org

# Author comment: Author response to Decision Letter 1

**DOI:** 10.1371/journal.pone.0332798.r002

6 Aug 2025

Response to reviewers

Reviewer 1:

Comment 1 - Lack of Experimental Validation: While the computational results are promising, the manuscript would benefit from a clearer discussion of the next steps for in vitro/in vivo validation, especially given that predictions can sometimes fail to translate biologically.

Response – Dear Sir, I appreciate your valuable suggestion. To address this point, I have expanded the Discussion section to include a specific paragraph highlighting the need for experimental validation.

Comment 2 - It is not entirely clear how novel the top three compounds are in terms of chemical scaffolds compared to known Tankyrase inhibitors. A comparison with structurally similar known inhibitors would strengthen the novelty claim.

Response – Thank you for the valuable comment. A scaffold-level comparison has been added to clarify the novelty of the top compounds.

Comment 3 - More details are needed on how the ML model was selected and validated, particularly the choice of descriptor types and hyperparameters for the final model.

Response - I thank you for the suggestion. The Methods section has been revised to include details on descriptor types, model selection, hyperparameter tuning, and validation strategy.

Comment 4 - Some sections, particularly the MD simulation and interaction discussion, are repetitive and could be condensed without loss of clarity.

Response – I thank you for the observation. The MD simulation and interaction sections have been revised to remove redundancy and improve clarity.

Reviewer 2:

Comment -1 Excellent integration of multiple computational techniques: DFT + MD + ML + Docking. Logical flow from database screening to MD validation and predictive modeling. But you need to add experimental or literature-based activity values for validation of the ML predictions. Include visual summaries or flowcharts for the workflow (database → docking → DFT → MD → ML).

Discuss limitations of the computational approach (e.g., force field limitations, ML data size, docking rigidity). Consider using ADMET profiling or synthetic accessibility scores to prioritize the hits.

Response – Dear Sir, I thank you for the positive feedback and constructive suggestions. I have added literature-based activity values to support ML predictions and included a workflow diagram summarizing the computational pipeline. Limitations of the approach and comments on ADMET profiling and synthetic accessibility have been addressed in the revised Discussion section.

Reviewer 3:

*The authors of the provided manuscript explored the potential anti-cancer activity of PubChem-deposited small molecule inhibitors of tankyrase biotarget. Comprehensive in silico study was performed over multi-stage screening stage to identify the potential hits. The manuscript is relevant in the field of drug discovery. Comments are to be addressed as follows:

Response - I would like to thank you for your thoughtful comments and constructive feedback. I appreciate the time and effort taken to review my manuscript, and I am grateful for your recognition of the relevance of my work in the field of drug discovery. I have carefully addressed the comments provided to improve the manuscript further.

Comment 1 - In the molecular modelling simulation section, brief description regarding the tankyrase’s topology, binding site description, and key binding residues being highlighted important within the current literature should be provided.

Response - I have added a brief description of tankyrase’s domain architecture, binding site features, and key residues at the beginning of the Molecular Dynamics Simulation section to clarify the structural basis for ligand interaction.

Comment 2 - Further, the compounds’ polar interaction patterns with key pocket residues should be annotated in terms of both the bond distances and angles. Hydrogen bonding should be presented within hydrogen bond distances as well as bond angles since hydrogen bond depend on both. Authors should mention the Hydrogen bond angles as well as their distances, since the strength of hydrogen bonding is based on both parameters in a way to ensure the adequacy of optimum hydrogen bonding.

Response - Thank you for your suggestion. I have included both hydrogen bond distances and angles in the revised manuscript, with the detailed information provided in Supplementary Figure 1.

Comment 3 - The authors provided Free binding energy calculations out of the MD simulation trajectories. It is advised to provide the dissected energy terms of these total energies (ΔGbind Lipo, ΔGbind Solv GB, ΔGbind vDW, ΔGbind Coulomb, and ΔGbind Ligand strain) to further account for simulated solvation entropy and evaluate the nature of interaction (dominant energy potential) based on these different energy terms. This would guide further hit to lead and lead optimization steps.

Response – I sincerely thank you for this insightful suggestion. I fully agree that decomposing the binding energy would offer essential insights; however, I was unable to perform this analysis due to the lack of access to the required licensed software. I kindly request your understanding in this regard.

Comment 4 - Based on the study results, what are the take-away messages. Authors are advised to highlight the future suggested structural modifications that would improve the hits’ predicted activities based on the computational findings. These insights would be beneficial for guiding future lead optimization and development.

Response - I thank you for the valuable suggestion. A summary of key interaction features and proposed structural modifications has been added to help guide future lead optimization and development.

Attachment Submitted filename: Response to reviewers.docx

# Peer review: Decision Letter 1

**DOI:** 10.1371/journal.pone.0332798.r003

**Authors:** Ahmed Al-Karmalawy

4 Sep 2025

Structural Stability-Guided Scaffold Hopping and Computational Modeling of Tankyrase Inhibitors Targeting Colorectal Cancer

PONE-D-25-34311R1

Dear Dr. Alshahrani,

We’re pleased to inform you that your manuscript has been judged scientifically suitable for publication and will be formally accepted for publication once it meets all outstanding technical requirements.

Within one week, you’ll receive an e-mail detailing the required amendments. When these have been addressed, you’ll receive a formal acceptance letter and your manuscript will be scheduled for publication.

An invoice will be generated when your article is formally accepted. Please note, if your institution has a publishing partnership with PLOS and your article meets the relevant criteria, all or part of your publication costs will be covered. Please make sure your user information is up-to-date by logging into Editorial Manager at Editorial Manager®  and clicking the ‘Update My Information' link at the top of the page. For questions related to billing, please contact billing support .

If your institution or institutions have a press office, please notify them about your upcoming paper to help maximize its impact. If they’ll be preparing press materials, please inform our press team as soon as possible -- no later than 48 hours after receiving the formal acceptance. Your manuscript will remain under strict press embargo until 2 pm Eastern Time on the date of publication. For more information, please contact onepress@plos.org.

Kind regards,

Ahmed A. Al-Karmalawy, PhD

Academic Editor

PLOS ONE

Additional Editor Comments (optional):

Reviewer #1:

Reviewer #3:

Reviewers' comments:

Reviewer's Responses to Questions

**Comments to the Author**

Reviewer #1: All comments have been addressed

Reviewer #3: (No Response)

**********

2. Is the manuscript technically sound, and do the data support the conclusions?

Reviewer #1: Yes

Reviewer #3: (No Response)

**********

3. Has the statistical analysis been performed appropriately and rigorously?

Reviewer #1: Yes

Reviewer #3: (No Response)

**********

4. Have the authors made all data underlying the findings in their manuscript fully available?

The PLOS Data policy

Reviewer #1: Yes

Reviewer #3: (No Response)

**********

5. Is the manuscript presented in an intelligible fashion and written in standard English?

Reviewer #1: Yes

Reviewer #3: (No Response)

**********

Reviewer #1: (No Response)

Reviewer #3: (No Response)

**********

what does this mean? ). If published, this will include your full peer review and any attached files.

If you choose “no”, your identity will remain anonymous but your review may still be made public.

**Do you want your identity to be public for this peer review?** For information about this choice, including consent withdrawal, please see our Privacy Policy

Reviewer #1: No

Reviewer #3: **Yes**

**********

# Editor report: Acceptance letter

**DOI:** 10.1371/journal.pone.0332798.r004

**Authors:** Ahmed Al-Karmalawy

PONE-D-25-34311R1

PLOS ONE

Dear Dr. Alshahrani,

I'm pleased to inform you that your manuscript has been deemed suitable for publication in PLOS ONE. Congratulations! Your manuscript is now being handed over to our production team.

At this stage, our production department will prepare your paper for publication. This includes ensuring the following:

* All references, tables, and figures are properly cited

* All relevant supporting information is included in the manuscript submission,

* There are no issues that prevent the paper from being properly typeset

You will receive further instructions from the production team, including instructions on how to review your proof when it is ready. Please keep in mind that we are working through a large volume of accepted articles, so please give us a few days to review your paper and let you know the next and final steps.

Lastly, if your institution or institutions have a press office, please let them know about your upcoming paper now to help maximize its impact. If they'll be preparing press materials, please inform our press team within the next 48 hours. Your manuscript will remain under strict press embargo until 2 pm Eastern Time on the date of publication. For more information, please contact onepress@plos.org.

You will receive an invoice from PLOS for your publication fee after your manuscript has reached the completed accept phase. If you receive an email requesting payment before acceptance or for any other service, this may be a phishing scheme. Learn how to identify phishing emails and protect your accounts at https://explore.plos.org/phishing.

If we can help with anything else, please email us at customercare@plos.org.

Thank you for submitting your work to PLOS ONE and supporting open access.

Kind regards,

PLOS ONE Editorial Office Staff

on behalf of

Associate Professor Ahmed A. Al-Karmalawy

Academic Editor

PLOS ONE
